# Guidelines for bioinformatics of single-cell sequencing data analysis in Alzheimer’s disease: review, recommendation, implementation and application

**DOI:** 10.1186/s13024-022-00517-z

**Published:** 2022-03-02

**Authors:** Minghui Wang, Won-min Song, Chen Ming, Qian Wang, Xianxiao Zhou, Peng Xu, Azra Krek, Yonejung Yoon, Lap Ho, Miranda E. Orr, Guo-Cheng Yuan, Bin Zhang

**Affiliations:** 1 Department of Genetics and Genomic Sciences, Icahn School of Medicine at Mount Sinai, 1470 Madison Avenue, Room S8-111, New York, NY 10029 USA; 2 Mount Sinai Center for Transformative Disease Modeling, Icahn School of Medicine at Mount Sinai, 1470 Madison Avenue, Room S8-111, New York, NY 10029 USA; 3 Institute for Personalized Medicine, Icahn School of Medicine at Mount Sinai, One Gustave L. Levy Place, New York, NY 10029 USA; 4 Department of Internal Medicine, Section of Gerontology and Geriatric Medicine, Wake Forest School of Medicine, Winston-Salem, North Carolina USA; 5 Sticht Center for Healthy Aging and Alzheimer’s Prevention, Wake Forest School of Medicine, Winston-Salem, North Carolina USA; 6 Icahn Institute of Genomics and Multiscale Biology, Icahn School of Medicine at Mount Sinai, 1470 Madison Avenue, Room S8-111, New York, NY 10029 USA; 7 Department of Pharmacological Sciences, Icahn School of Medicine at Mount Sinai, 1470 Madison Avenue, Room S8-111, New York, NY 10029 USA

**Keywords:** Alzheimer’s disease, Single cell sequencing, Single cell RNA-sequencing, Single cell ATAC-sequencing, Spatial transcriptomics, Clustering analysis, Trajectory analysis, Gene networks, And brain cell types

## Abstract

Alzheimer’s disease (AD) is the most common form of dementia, characterized by progressive cognitive impairment and neurodegeneration. Extensive clinical and genomic studies have revealed biomarkers, risk factors, pathways, and targets of AD in the past decade. However, the exact molecular basis of AD development and progression remains elusive. The emerging single-cell sequencing technology can potentially provide cell-level insights into the disease. Here we systematically review the state-of-the-art bioinformatics approaches to analyze single-cell sequencing data and their applications to AD in 14 major directions, including 1) quality control and normalization, 2) dimension reduction and feature extraction, 3) cell clustering analysis, 4) cell type inference and annotation, 5) differential expression, 6) trajectory inference, 7) copy number variation analysis, 8) integration of single-cell multi-omics, 9) epigenomic analysis, 10) gene network inference, 11) prioritization of cell subpopulations, 12) integrative analysis of human and mouse sc-RNA-seq data, 13) spatial transcriptomics, and 14) comparison of single cell AD mouse model studies and single cell human AD studies. We also address challenges in using human postmortem and mouse tissues and outline future developments in single cell sequencing data analysis. Importantly, we have implemented our recommended workflow for each major analytic direction and applied them to a large single nucleus RNA-sequencing (snRNA-seq) dataset in AD. Key analytic results are reported while the scripts and the data are shared with the research community through  GitHub. In summary, this comprehensive review provides insights into various approaches to analyze single cell sequencing data and offers specific guidelines for study design and a variety of analytic directions. The review and the accompanied software tools will serve as a valuable resource for studying cellular and molecular mechanisms of AD, other diseases, or biological systems at the single cell level.

**Supplementary Information:**

The online version contains supplementary material available at 10.1186/s13024-022-00517-z.

## Background

Alzheimer’s disease (AD) is one of the most devastating forms of dementia common in the elderly, estimated to affect over 6.2 million individuals in the United States and 24 million worldwide [1, 2]. Clinically, AD patients present amnestic multidomain progressive dementia. A more definitive AD diagnosis requires evidence of amyloid-beta (Aβ) plaques and Tau neurofibrillary tangle (NFT) accumulation within the neurodegenerative brain [3].

AD is a highly complex and heterogeneous disease caused by various pathophysiologic mechanisms. AD can be classified by heritable cause and age of onset, i.e.*,* rare familial AD, sporadic early-onset (EOAD), and late-onset (LOAD) [4]. While AD often progresses through a period of mild cognitive impairment (MCI), not all patients with MCI develop AD, hinting at protective or causal factors that may differentially affect subsets of patients even within traditional subtypes. Postmortem evaluations revealed that AD brains may include depositions of additional pathologies (i.e.*,* beyond Aβ and phosphorylated tau), such as Lewy bodies, alpha-synuclein, transactive response DNA-binding protein and/or vascular-related brain lesions [5]. Further, the recently discovered five molecular subtypes of AD were associated with unique molecular signatures and distinct sets of brain cell type-specific key regulators [6].

**Fig. 1:**
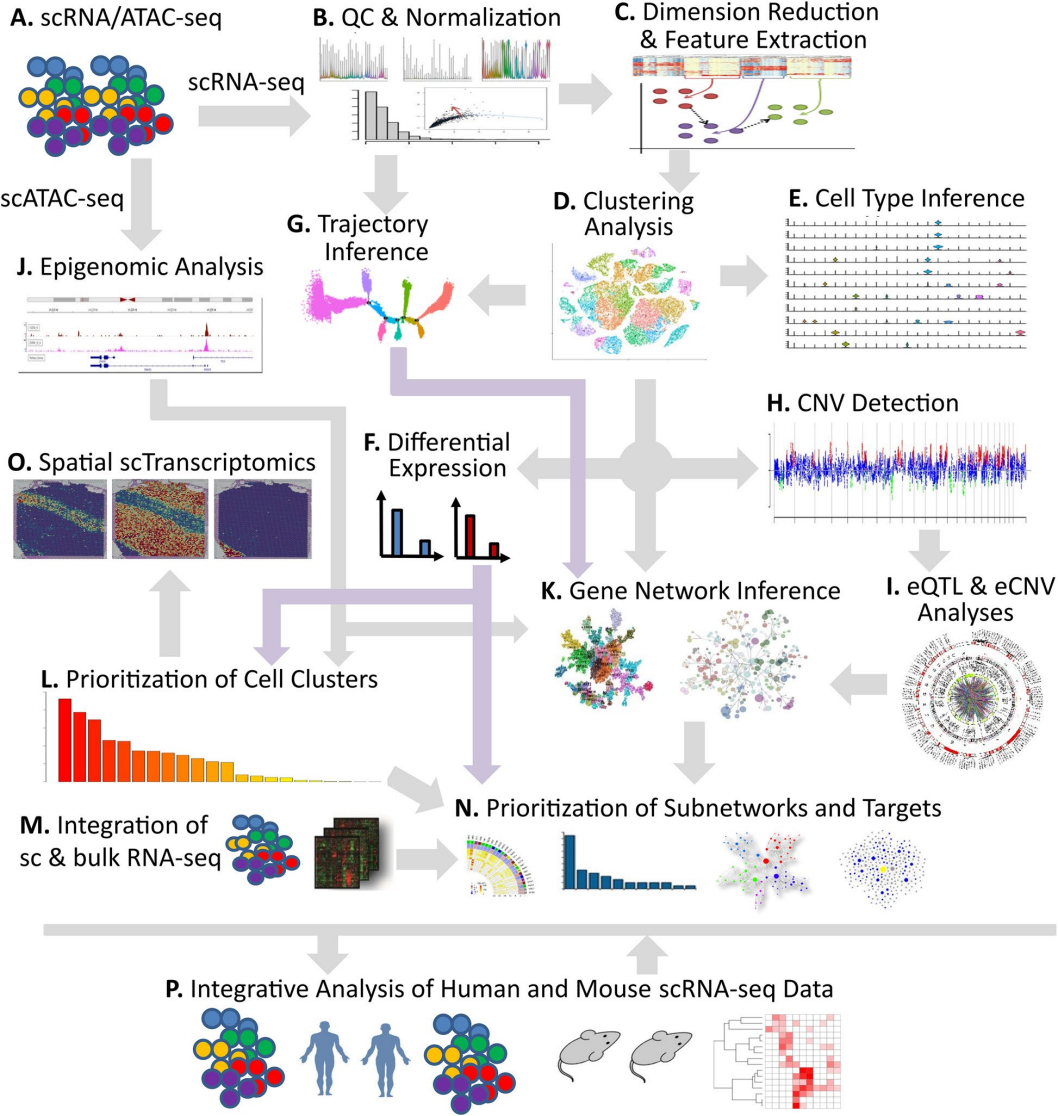
Overview of the bioinformatics approaches to analyze scRNA-seq, scATAC-seq, and spatial transcriptomics data with a focus on scRNA-seq data. scRNA-seq and scATAC-seq data (**A**) go through appropriate quality control (QC) to remove outliers and cells with low-quality sequencing data (**B**), followed by normalization (**B**). QC-ed and normalized data are then used for dimension reduction, and feature extraction (**C**) clustering analysis to identify cell clusters (**D**). Marker genes for each cell cluster will then be identified to infer its association to known or novel cell type (**E**). Meanwhile, differential gene expression is performed between cell groups of interest (e.g., AD and Control) in each cell cluster to identify gene expression changes associated with the disease (**F**). Trajectory inference can be performed on all cells, cells in each cluster or the cells from multiple closely related cell clusters to infer cellular dynamics during developmental or disease progression (**G**). Copy number variations (CNVs) can also be inferred from scRNA-seq data (**H**). Integration of gene expression and genomic (SNPs & CNVs) data leads to the identification of expression-associated quantitative trait loci (eQTLs) (**I**). Epigenomic analysis by scATAC-seq can study gene expression regulatory elements in open chromatin regions (**J**) and will be detailed in Fig. 7. Gene coexpression and causal networks will be constructed for each cell cluster or multiple closely related cell clusters, while priors from eQTLs and epigenomic analyses can be developed for assisting causal network inference (**K**). Cell clusters can be prioritized based on the number of differentially expressed genes between disease and control across all cell clusters (**L**). scRNA-seq data can also be integrated with bulk RNA-seq data to robustly identify key molecular changes and network structures (**M**). Finally, cell cluster-based networks will be analyzed to prioritize key subnetworks (e.g., coexpressed gene modules) and potential network regulators for a disease (e.g., AD) under study (**N**). Novel cell clusters, key subnetworks and key driver genes can be validated through single-cell spatial transcriptomics analysis which offers more insights into spatially distributed molecular signals in a system or a disease under study (**O**). Key findings from human AD single cell sequencing data will be validated in AD mouse models and integration of mouse and human single cell data is critical for informing the correspondence between AD mouse models and human AD (**P**)

Understanding the cell-type-specific changes and regulations at the single-cell level will enable us to decode the molecular mechanisms underlying the pathophysiologic processes contributing to dementia. Indeed, recent single-cell sequencing studies of aged and AD brains revealed a series of brain cell clusters involved in AD [7, 8]. However, these studies primarily focused on clustering and differential analyses but did not fully exploit the single-cell sequencing data to explore, for example, pseudo-temporal dynamics. To mitigate these gaps, we reviewed the state-of-the-art bioinformatics approaches to analyze single-cell transcriptome (single-cell/−nuclei RNA sequencing (sc/snRNA-seq)) and epigenome (single-cell assay for transposase-accessible chromatic sequencing (scATAC-seq)) in AD, and integrate single-cell features with abundantly available AD bulk sequencing data. Specifically, we reviewed the following 15 topics (Fig. 1): 1) quality control and normalization, 2) dimension reduction and feature extraction, 3) cell clustering analysis, 4) cell type inference and annotation, 5) differential expression for disease gene identification, 6) trajectory inference, 7) copy number variation (CNV) analysis, 8) integration of single-cell multi-omics (e.g., expression associated quantitative trait loci (eQTL) and expression associated CNVs (eCNVs)), 9) epigenomic (scATAC-seq) analysis, 10) gene network inference, 11) prioritization of cell clusters, 12) integration of single cell and bulk RNA-seq data, 13) spatial single-cell transcriptomics, and 14) comparison between single cell AD mouse model studies and single cell human AD studies. For future directions, we discuss experimental validation strategies of single-cell based findings, and translations to drug discoveries. Notably, we implemented our recommended workflow for each major analytic direction and applied them to a large snRNA-seq dataset in AD. Key analytic results were reported while the scripts and the data were shared with the research community through GitHub (see the section "Availability of data and software code" for the details). We hope that the guidelines will accelerate AD research by leveraging the power of single-cell sequencing.

### Overview of single-cell sequencing study design

High-throughput sequencing of bulk tissue measures the average signals of various cell types, thus falls short of dissecting the cellular heterogeneity in brain tissues. To address this issue, single-cell sequencing has been recently developed to elucidate the cell-type specificity and identify the transcriptome, epigenome, and genome changes among various cellular populations. Recently, scRNA-seq studies have been widely conducted in many research fields, such as oncology [9], developmental biology [10], immunology [11], and neurosciences [12]. Several protocols have been developed to measure mRNAs and non-coding RNAs from single cells, such as Smart-seq [13], Quartz-seq [14], CEL-seq [15], RamDa-seq [16], Drop-seq [17], sci-RNA-seq [18] and Chromium (10X Genomics).

Single-cell genome and epigenome including single-cell ChIP-seq [19] and scATAC-seq have also emerged to investigate the genomic and epigenomic status associated with the transcriptome of cells [20, 21]. Single-cell genome sequencing captures de novo germline mutations, somatic mutations, and copy number alterations to dissect the genetic heterogeneity at the cellular level [22]. scATAC-seq is useful in analyzing the patterns of open chromatin, a hallmark of active regulatory elements, in single cells. Moreover, several advanced spatial sequencing techniques (for example, 10x Visium) have included spatial dimensions of the molecular features at a near-cellular resolution [23, 24]. Although the sequencing protocols have improved platforms and reagent kits to increase the detection sensitivity, the sequencing coverage is still limited, and present challenges to robustly analyzing single-cell sequencing data.

Several considerations should be taken into account for designing single-cell sequencing studies. The first one is tissue requirement. For example, whole single-cell RNA sequencing requires fresh samples, thus requiring the study to implement a seamless process from obtaining patients’ consent, acquiring the autopsy samples, to preparing a single-cell library even sequencing within a few hours [7, 25]. On the contrary, fresh frozen samples in a tissue repository can be preserved for a substantial amount of time, providing the freedom to select samples with relevant clinical, molecular characteristics such as gender and APOE genotype, and their nuclei can be isolated for snRNA-seq data analysis. The second consideration is cellular coverage. Several cell types, especially neurons, are underrepresented in scRNA-seq dataset, due to technical issues relating to size selection during the tissue dissociation process [26, 27], while snRNA-seq covers more cell types [28, 29]. However, in selecting single-cell or single-nuclei based approaches for transcriptomic profiling, it is important to recognize that each has its strengths and limitations. Brakken et al. compared these two approaches side by side by generating matched datasets from the mouse visual cortex [30]. They found that scRNA-seq analysis has the strengths of unbiased transcriptomic profiling, a higher gene coverage rate, and a higher-power for distinguishing similar cell types. However, the tissue dissociation and cell-isolation protocols are too harsh for certain cell types, leading to significant under-representation. In contrast, snRNA-seq has the strengths of less biased cellular coverage, resistance to cell isolation-associated perturbations, and applicability to both fresh and archived frozen specimens. Single-nuclei detected transcripts are also enriched for intronic reads, whereas the majority of the single-cell detected transcripts are from exons. Interestingly, the nuclear proportion of total cellular mRNA varies significantly in a cell-type and cortical-layer-specific manner, although the biological significance of such variation is still unknown. However, Thrupp et al. [31] found that snRNA-seq data is depleted of an activated microglial subpopulation expressing the activation signature, including *APOE, CST3, SPP1* and *CD74*, but the absence of the microglial subpopulation was likely due to the low sequencing depth. Despite these differences, it is important to note that the overall cell-type landscapes captured by these two approaches are similar [30]. Power analysis is a critical step to rationalize scRNA-seq study design to ensure robustness and reproducibility of scientific findings. In the companion GitHub repository (see the section "Availability of data and software code" for details), we provided a comprehensive review of the power analysis approaches for single cell studies and shared the script for applying a recommended approach to an AD snRNA-seq study. 

Different sequencing protocols are optimized for different biological aspects. PCR plate-based sequencing protocols (e.g. Smartseq2 [32], CEL-seq [15], and MARS-seq [33]) capture cells through cell sorter or microfluidics and offer high read depth per cell with less effective cell captures [34]. Thus, these protocols provide high sensitivity to discriminate subpopulations of similar cell types with subtle differences [35]. On the contrary, droplet-based protocols (e.g. InDrop [36], Drop-seq [17], and 10x Chromium [37]) capture thousands to millions of cells with low sequencing depths per cell [34], and can offer exogenous spike-ins to handle technical noises systematically [38, 39]. Large numbers of cells in these protocols enable the detection of rare cell populations such as neuronal subtypes [40]. However, Alsema et al. 2020 report that single-cell sequencing of FACS sorted microglia by droplet-based 10x Chromium and PCR plate-based Smart-seq2 only displayed marginal differences, most likely arising from technical noises by plate-based protocols [25]. These indicate targeted studies for cell type of interest may not require large-sequencing depths to uncover distinct sub-populations. So far, the droplet-based 10x Chromium snRNA-seq, which can sequence over 10,000 nuclei per library, is the most widely used sequencing platform for human cohort studies including AD (Table 1).

**Table 1:** Summary of study design and single-cell RNA sequencing platforms in various human cohort studies of AD. PMID: PubMed id

Study	PMID	Platform	Study design	Note
Grubman et al. 2019 [41]	31768052	10x isolated single-nuclei RNA sequencing	6 AD, 6 controls from tissue repository	
Mathys et al. 2019 [8]	31042697	10x isolated single-nuclei RNA sequencing	24 AD pathology, 24 no pathology from tissue repository	
Alsema et al. 2020 [25]	33192286	10x single-cell RNA sequencing and Smart-seq2	FACS sorted microglia from 27 autopsy samples within 6 h after death	10x scRNA-seq and Smart-seq2 showed some differences, mainly small clusters suspectedly due to plate-based protocols in Smart-seq2.
Lau et al. 2020 [42]	32989152	10x isolated single-nuclei RNA sequencing	12 AD and 8 control from tissue repository	Male and female ratio were balanced in AD and control
Nguyen et al. 2020 [43]	32840654	10x isolated single-nuclei RNA sequencing	15 AD from tissue repository	Samples were selected with varying APOE genotypes and pathologies, but matched for age and sex.
Gerrits et al. 2021 [44]	33609158	10x isolated single-nuclei RNA sequencing	10 AD	10 AD samples representing Braak stages 0, 2 and 6, all *APOE* ε3/ε3 genotypes
Morabito et al. 2021 [45]	34239132	10x isolated single-nuclei RNA sequencing and ATAC sequencing	12 AD prefrontal cortex (PFC), 8 control PFC	
Olah et al. 2020 [7]	33257666	10x single-cell RNA sequencing	FACS sorted microglia from 10 AD, 4 Mild Cognitive Impairment and 3 temporal lobe epilepsy samples in DLPFC	

### Quality control and normalization

Data quality control (QC) and normalization are the essential steps to remove systematic sources of technical variations introduced during the single-cell data generation process while preserving the true biological variations. Due to the low amount of RNA in a single cell and the stochastic sampling process of sequencing, scRNA-seq data are much noisier than bulk-tissue sequencing data [46, 47]. Excessive zero or near-zero counts by the so-called “dropout” events [48], often lead to highly sparse data, shadow the biological variations in individual cells and require dedicated QC metrics to ensure that only high-quality data are selected for downstream analysis. Starting from a count matrix of unique molecular identifiers (UMIs), a typical data preprocessing workflow generally contains several steps for QC to remove low-quality cells and genes, and normalize cell-specific biases.

#### Quality control on the cells

Two common quality measures are the number of expressed features (i.e., features detected with non-zero counts) and the library size (i.e., the sum of counts across all features). Violin plots are used to visualize the distribution of these cell-specific measures in each donor sample [49, 50]. Cells with very few expressed features or small library size indicate low RNA-capture efficiency and are hence considered poor quality. On the other hand, cells with abnormally a large number of expressed features suggest doublets or multiplets (i.e., two or more cells mistakenly captured as a single cell) [51], hypothesizing that doublets or multiplets would have higher total RNA content (see below for a review of more elegant doublet detection methods). Thus, a lower and an upper bound for the number of expressed features can be specified for cell filtering. However, determining the bounds for the number of expressed features or library size is not trivial as both biological and technical factors need be taken into account. For example, sequencing with deeper depth leads to more reads and more expressed features, irrespective of the cell quality. Another filtering approach is to detect outliers. For instance, it has been proposed to remove cells with log-library size greater than 3 median absolute deviations (MADs) or below the median log-library size [52, 53].

The presence of doublets or multilets may severely confound the downstream analysis and interpretation. This can lead to, for example, spurious cell clusters, both false positive and false negative prediction of cell cluster markers or disease genes, biased cell-state trajectories, and misrepresented gene-gene correlation structure and gene regulatory networks [54–56]. Doublet detection can be facilitated through appropriate experimental design. These include species mixing (mixing of cells from different species), mixing of cells from samples with different genotypes or genetic labels, and cell “hashing” (pooling of cells from separately barcoded samples) (see [55] for a summary of the experimental assay-based methods, including their limitations, for doublet detection). However, most of the existing AD single-cell datasets have not implemented the experimental design features. This review will focus on the model-based doublet detection approaches that are applicable to all AD scRNA-seq datasets currently available.

Assuming that doublets have more RNAs than singlets, the simplest approach is to threshold overall expression content (such as the number of detected genes and total UMI counts) to classify cells with unusually high UMI or gene number as potential doublets [51, 55]. However, the assumption that cells contain similar amount of RNA is unlikely to be true due to diverse cell types or different cell cycle states. Another simple approach is to look for cells expressed with marker genes of more than one distinct cell type [51, 55, 57]. However, this requires expert knowledge of the cell types and the associated markers in the data. There are more advanced and potentially more powerful computational algorithms for doublet detection in scRNA-seq data. In a recent benchmarking study, Xi and Li evaluated nine existing doublet detection methods [54], including Scrublet [55], scran/doubletCells [58], cxds [56], bcds [56], hybrid (combination of cxds and bcds) [56], DoubletDetection [59], DoubletFinder [60], Solo [61], and DoubletDecon [62]. Seven out of the eight standalone methods (except cxds which detects co-expression of markers that are supposedly to be mutually exclusive in the same cell) first generate artificial doublets by mixing observed gene expression profiles from randomly selected droplet pairs. The major difference among these methods is the choice of embedding/dimension reduction and classifier. Via a comprehensive benchmarking on 16 real datasets with experimentally annotated doublets and 112 realistic synthetic datasets, DoubletFinder showed the best prediction accuracy while cxds had the highest computational efficiency. However, one caveat of these computational algorithms is that they were designed to identify “neotypic” doublets, which consist of cells of distinct cell types, and hence difficult to capture “embedded” doublets that encapsulate cells from the same or highly similar cell types [55].

An important cell quality measure is the percentage of reads mapped to the mitochondrial genome in each library. As increased mitochondrial fraction indicates increased apoptosis, increased cell stress, and/or loss of cytoplasmic RNA from lysed cells [52, 63], cells with a high proportion of reads allocated to mitochondrial genomes are deemed poor-quality. A recent systematic survey of scRNA-seq data suggested that a mitochondrial proportion threshold of 10% is appropriate to distinguish between healthy and low-quality cells in most human tissues, while in mouse tissues, the recommended threshold is 5% [64]. However, just like the number of expressed features, selection of a threshold for this parameter is highly dependent on tissue type and experimental setting. For example, 30% of mitochondrial mRNA, which would otherwise indicate cell stress or apoptosis in tissues with low energy need, is normal for a healthy heart muscle cell due to high energy demand [65, 66]. Mitochondrial transcripts are not expressed in nuclei. Yet, variable amounts of mitochondrial transcripts were associated with the snRNA-seq data [8, 41, 42, 67, 68]. For example, in the first snRNA-seq transcriptomic analysis of AD, the fraction of mitochondrial reads exhibited a highly skewed empirical distribution, with an elbow shape which distinctly separates cells with high and low ratios for further classification and removal by k-means clustering (k = 2) on the mitochondrial ratio [8].

Another source of noises in the droplet-based scRNA-seq protocols (e.g., drop-seq or 10x Genomics Chromium protocol) is the contamination of ambient RNAs (cell-free RNAs), which are released in the cell lysis from dead or apoptotic cells before droplet separation. As ambient mRNAs are ubiquitous, they increase background noise and may significantly confound data quality and biological signal [69]. Several methods have been developed to remove the contribution of the ambient RNAs from each cell to recover the true molecular abundance. For example, the SoupX method estimates the ambient mRNA expression profile from empty droplets and the contamination fraction in each cell by making use of known negative cell markers in an identified cell cluster, and then corrects the expression of each cell using the two paratemers [69]. There are other ambient RNA decontamination methods that do not require prior knowledge of negative cell markers, such as DecontX [70] which uses a Bayesian inference model to estimate and remove the background noise, and CellBender [71] which employs a deep generative model to remove the background noise from ambient RNA. In the mixed-sample multiplexing scRNA-seq design, where multiple samples of different genotypes are pooled, or in the presence of subclones, a method called Souporcell can demultiplex cells, identify doublets, and perform joint genotyping and ambient RNA amount estimation by modeling the allele counts of genetic variants available from the reads [72]. Ambient RNA detection and removal is an emerging area of research and just began to be included in the AD snRNA-seq studies [44, 73]. However, since the leak of cytoplasmic RNA by ruptured cells to the cell suspension is unavoidable by the isolation protocols, especially for the case of nuclei isolation from fresh frozen tissues, we expect incorporating the ambient RNA decontamination into the single cell data analysis pipeline will provide much cleaner downstream analysis in future applications of AD.

#### Quality control and filtering on genes

Genes with low abundance should be removed since they do not contain sufficient information for reliable downstream statistical analysis [74]. Thresholds can be set for the number of cells expressing a gene or the mean expression of a gene [52]. The cell number threshold could be very liberal (e.g., 2 cells in some of the published brain disease studies [8, 68]). Still, it is critical not to exceed the minimal cell cluster size that one may reasonably expect [75].

Further, depending on the downstream analysis, some feature categories such as non-coding genes may not be of interest and hence could be removed to reduce the data complexity [8]. Mitochondrially expressed genes can be also discarded after cell QC in snRNA-seq data to avoid biases introduced during the nuclei isolation since mitochondrial transcripts are not expressed inside a nucleus [8, 67, 68].

#### Normalization

The observed single-cell read count data could be impacted by many biological and technical factors, including but not limited to sequencing depth, capture efficiency, and cell composition. Between-sample normalization can remove these sample-specific biases and mitigate the batch effect. The simplest method is scaling normalization, which corrects for sequencing depth difference by dividing the feature-level read counts by the library size (i.e., total read counts within each sample) and multiplying a constant value (e.g., 10,000). The library size corrected data is usually log-transformed after adding value 1 to prevent the logarithm of 0. This normalization strategy is implemented in popular tools like scanpy [50] and Seurat [76]. However, similar to bulk RNA-seq normalization, library size as a scaling factor is likely to bias towards highly expressed transcripts. In the context of bulk RNA-seq, there are three most popular methods for robust scaling normalization, including 1) the trimmed mean of M-values (TMM) [77], which calculates scaling factors by trimming away genes with extreme fold changes between samples; 2) the upper-quartile (UQ) method [78], which uses per-sample upper-quartile (75-th percentile) to scale counts; 3) the relative log-expression (RLE) [79], which scales to a pseudo-reference derived from the geometric mean of gene counts across cells.

While these bulk-based methods are still widely used in scRNA-seq data [74, 80], the excessive zeros in the scRNA-seq data jeopardize their effectiveness in calculating proper scaling factors. For example, the TMM method tends to overcorrect for the scaling factors [80] and the upper-quartile could be zero for many cells with low sequencing depth. Moreover, calculating the pseudo-reference sample from the geometric mean across cells can be applied to only the potentially minimal number of genes with non-zero reads in every single cell [80]. Alternatively, the dropout reads can be imputed by assuming a mixture model that includes two latent probability distributions: the probability of the true expressed reads and dropout reads among the true expressed reads. These model-based methods include SAVER [81] (Poisson-Gamma mixture model) and scImpute (Normal-Gamma mixture model) [82]. Markov Affinity-based Graph Imputation of Cells (MAGIC), on the other hand, utilizes a diffusion kernel to identify similar cells in reduced dimension, and infer the dropout reads from the similar cells [83].

Several scRNA-seq-specific normalization methods have been developed and they can be primarily classified into two categories: 1) cell-based normalization by estimating a cell-specific global size factor to normalize all the genes in the same cell, and 2) gene-based normalization by parametric modeling of individual genes. The scran package adopts the cell-based normalization approach by pooling the cells to estimate more robust size factors and avoid the impact of excessive zeros. Then pool-based size factors are “deconvolved” to yield cell-specific factors [84]. In contrast, the gene-based normalization methods, such as the SCnorm [85] and the Pearson residuals method SCTransform in the Seurat package [76], perform adjustments individually for each group of genes with different sequencing depths or different ranges of abundance levels. In addition to the correction for sequencing depth bias for different groups of genes, parametric modeling of count data can account for more complex technical or biological variations, such as batch effect, mitochondrial transcript fraction, cell cycle effect, cellular detection rate (fraction of detected genes), and the average number of counts per detected genes [86–89]. Moreover, cell-level and gene-level variations can be jointly modeled in a unified framework. For instance, for better separation of unwanted variation from biological signals in noisy, zero-inflated scRNA-seq data, Risso et al. proposed a Zero-Inflated Negative Binomial-based Wanted Variation Extraction (ZINB-WaVE) method, which incorporates not only observed and unobserved sample-level but also gene-level covariates (e.g., sequence length and GC content) [90].

Unwanted sources of variations such as batch effects should be adjusted using single-cell dedicated tools (e.g., MNN [91], CCA [92]), or general linear regression modeling tool (e.g., limma [93], ComBat [94]). Deep learning-based data denoising tools such as deep count autoencoder (DCA) [95] and single-cell variational inference (scVI) [96] are also attractive alternatives to handle unwanted variations in scRNA-seq.

#### Recommended workflow and application to AD

**Fig. 2:**
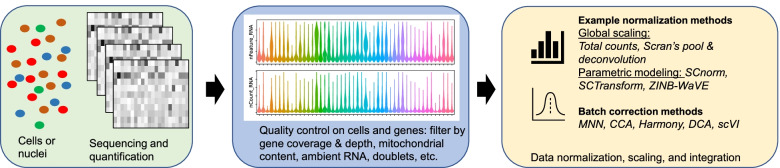
A workflow of sc/snRNA-seq data preprocessing. After obtaining the single cell or single nucleus sequencing count data, a series of quality control processes are conducted to filter low quality cells with unusually high or low gene coverage or sequencing depth, unusually high mitochondrial content, ambient RNA, and doublets etc. QCed count data is normalized by either a global scaling approach or advanced parametric modeling of the zero-inflated count data distribution. If there exists batch or condition-specific clustering of the cells, a data integration method like MNN and CCA can be used to correct the batch difference to ensure that cells of the same cell type cluster together

Figure 2 illustrates a workflow of preprocessing sc/snRNA-seq data, i.e., data QC and normalization. For data QC, we recommend to first inspect the distribution of cell-level read count statistics, such as the total number of UMI counts, the number of detected genes, and the percentage of mitochondrial reads. If no sample presents dramatically different data quality, we expect to see similar cell-level data distribution across donors. Otherwise, we should check if the sample data quality difference is associated with any biological or technical variable. Then confounding technical variables could be taken into account in the data normalization. For example, Seurat’s SCTransform normalization approach has an option “vars.to.regress” to regress out confounding factors. To investigate if any particular variables play significant contribution to the cell-level gene expression variation, mixed model variance component analysis (such as implementation by the R package variancePartition [97]) can be used to quantify the variance attributable to individual factors. For data normalization, we recommend evaluating several different methods. For example, start with the simplest approach of the global scaling by sequencing depth, and proceed to clustering and differential expression analysis (Sections C, D, and E). Then compare the clustering results of the simple method with those from more advanced/complex methods. We favor the methods that lead to better separation of cell clusters with clear cell type annotations and biological meaningful signatures. In cases of combining multiple batches (or conditions) of single cell data where there is batch (or condition) specific cell clustering, an elegant integration method such as Seurat/CCA [92] and harmony [98] should be used after normalization of individual datasets to minimize the batch difference.

In the first snRNA-seq analysis of control and AD brains by Mathys et al. [8], nuclei with fewer than 200 detected genes or an abnormally high ratio of mitochondrial reads were removed. Mitochondrially encoded genes were removed and only protein-coding genes detected in at least 2 nuclei were selected. In AD snRNA-seq study by Zhou et al. [57], they selected the nuclei with no more than 5% mitochondrial reads, 400–20,000 UMIs and 400–7000 genes as determined by UMI/gene distribution. In another AD snRNA-seq study by Grubman et al., the nuclei with more than 10% of their UMIs assigned to mitochondrial genes or nuclei outside the 5th and 95th percentiles in the number of detected genes or the number of UMIs were filtered out [41]. Similarly, AD snRNA-seq data analyses by Nguyen et al. [43], Lau et al. [99], and Gerrits et al. [44] also QCed their data by mitochondrial content and read count cutoffs, albeit with slightly different threshold values. In addition, Gerrits et al. conducted ambient RNA and cytoplasmic RNA identification to recover more cells from the raw data. In all these AD snRNA-seq studies, QCed data were normalized by the total library size multiplied by a factor of 10,000, except in the Zhou et al. study where they further regressed the total number of UMIs by a negative binomial model.

### Feature selection and dimension reduction

Dimension reduction is to identify a few latent variables that explain the most variance in data. Selecting the most informative gene features can improve the detection efficiency and quality of the latent variables. The criteria for selecting informative genes include high biological variances with which the technical variance is modeled by the fitted relationship between mean and variance or spike-ins [74, 76, 84], and strong correlations with different cell types [17, 92] or known pathways (PAGODA) [100].

Then, the latent variables underlying the informative genes are identified by various techniques. Principal Component Analysis (PCA) is an efficient linear algorithm applicable to large-scale matrices and preserves both local and long-range structures. Each principal component is an orthogonal vector to the rest, and their linear combinations can reconstruct the global transcriptome. The PCA dimension can be determined by selecting top PCs accounting for 80 ~ 90% of total variances, PCs with significantly higher loading than bootstrapped data, or detecting an elbow point in the PC loading plot [92]. Several PCA variants have emerged to handle drop-out reads via zero-inflated negative binomial distribution (ZINB) [90].

t-distributed stochastic neighbor embedding (t-SNE) is a non-linear approach to preserve the local structures in the high-dimensional data [101]. Due to the emphasis on the local structure, t-SNE has gained popularity for effectively segregating clusters, but loses long-range structures [34]. Diffusion map (DM) is another popular non-linear method that projects both local and long-range structures to a lower dimension and is optimized to trace gradual changes in a transcriptome [102]. However, DM and t-SNE are computationally expensive. Recently, a computationally more scalable method, uniform manifold approximation, and projection (UMAP), has been proposed to include more long-range structures than t-SNE [103]. UMAP shows superior performances in segregating local clusters than t-SNE while recovering some of the global structures in scRNA-seq data [103]. The non-linear projection methods (DM, t-SNE, UMAP) can be applied directly to the transcriptome or the PCs of interest to compress the data in 2 or 3 dimensions. It is worth noting that, however, they may distort the overall data structure and introduce non-biological artifacts [104] and are often recommended for visualization purposes explicitly. Overall, dimension reduction is useful for visual inspections of cell-level patterns in AD. After data quality control and selection of highly variable genes with, for instance, significant dispersions (FDR < 0.05), cells often aggregate into clusters with distinct molecular characteristics. Such patterns define not only cell types with respective marker gene expressions in the reduced dimensions but also heterogeneous distributions of cells in AD samples in contrast with those from healthy control ones.

### Unsupervised cell clustering analysis

Unsupervised cell clustering is a data-driven process to group cells that share similar molecular patterns in scRNA-seq reads. As this is an “unsupervised” approach, it minimizes the impact of external bias and serves to provide biological insights to understand distinct cell populations in the tissue of interest [34, 105].

The cell clustering approaches can be categorized into gene expression-based and genotype-based approaches. Gene expression-based approaches regard each cluster as a unique cell type or sub-population of a known cell type with a distinct expression pattern. However, the high-dimensionality of single-cell transcriptome incurs the “curse of dimensionality”, enforcing distances among the homogeneous cells and making it impossible to distinguish distinct cell populations [34]. This often necessitates dimension reduction before the clustering analysis. Once clusters are identified, the uniquely expressed genes in each cluster can serve as de novo markers to pinpoint, if any, the associated cell type and identify pathways underlying them [74, 92]. Further, gradual expression changes among these clusters may indicate temporal cellular dynamics to infer cell trajectory [106] or sub-clonal evolution [107].

On the other hand, genotype-based cell clustering approaches utilize sequencing reads to identify single nucleotide variants (SNVs) in individual cells and group the cells bearing a similar set of SNVs. The resulting cell clusters can be utilized to demultiplex the reads for individuals with distinct genotypes [108–110], identify clonal populations [107], or screen doublets and ambient RNA contamination [72]. We have curated the overview of the single-cell clustering tools in Table 2.

**Table 2:** Summary of the clustering analysis approaches for scRNA-seq data

Method	Clustering strategy	Dimension reduction	Similarity	Notes
***Expression-based***				
**SC3** [111]	consensus k-means in multiple similarity matrices	PCA	Euclidean distance, Spearman’s correlation, Pearson’s correlation	Joint calculation of multiple similarity matrices increases the computational burden
**SIMLR** [112]	A Gaussian kernel is jointly learned on Euclidean and Spearman’s correlations to infer block structure in cell-cell similarity.	t-SNE on learned cell-cell similarity	Euclidean distance, Spearman’s correlation, Pearson’s correlation	Searches for consensus block structures in multiple similarities
**DBSCAN** [113]	density-based clustering	user choice (usually t-SNE is preferred)	NA	Results may vary due to the stochasticity of t-SNE
**PhenoGraph** [114]	k-nearest neighbor graph	NA	Jaccard index, Euclidean distance	Jaccard index is used to prune spurious links. GN modularity is optimized by Louvain’s algorithm
**SNN-Cliq** [115]	shared k-nearest neighbor graph	NA	Euclidean distance	Maximal clique search is performed for small cliques. Quasi-cliques connecting the detected maximal cliques are further detected to identify dense subnetworks.
**MetaCell** [116]	k-nearest neighbor graph	NA	Pearson’s correlation	A series of regularizations are performed to construct a balanced, symmetrized, and weighted graph. This is followed by a variant k-means search in the graph.
**scvis** [117]	Model-based deep generative modeling to train deep neural network-based model	Deep neural network-based	NA	Log-likelihood of noise model serves as the loss to train a deep auto-encoder-based model.
**scVI** [96]	Model-based deep generative modeling to train deep neural network-based model	Deep neural network-based	NA	Similar to scvis. Additional noise parameters for dropout reads by ZINB and library sizes as Gaussian noises.
**DESC** [118]	Neural network based dimension reduction + Louvain’s method-based iterative clustering.	Deep neural network-based	NA	Autoencoder learns cluster-specific gene expressions, and handles technical variances (e.g. batch effects) when they are smaller than biological variances. GPU enabled to scale up for millions of cells. Combination of Louvain’s clustering and t-distribution based cluster assignment refines the clusters iteratively in the bottleneck layer.
***Genotype-based***				
**demuxlet** [110]	supervised clustering of cells based on genotypes	NA	NA	likelihood of cell belonging to an individual is calculated based on alternate allele frequency
**Vireo** [108]	supervised clustering of cells based on genotypes	NA	NA	variational Bayesian inference allows estimation on the number of unique individuals with distinct genotypes. Cells are assigned to the individual with maximum likelihood
**scSplit** [109]	unsupervised clustering of cells based on allele fraction model	NA	NA	Expectation-Maximization (EM) optimization of Allele Fraction model to probability of observing alternate alleles from individuals.
**Souporcell** [72]	mixture modeling	NA	NA	minimap2 instead of STAR aligner to optimize variant calling in scRNA-seq reads. The mixture model is fitted in the allele fraction model to perform clustering in genotype space.
**DENDRO** [107]	phylogeny reconstruction based on genetic divergence in cells	NA	NA	Intended for tumoral heterogeneity. Genetic divergence is modeled with nuisance variables such as dropout rates and library sizes.

#### Expression-based clustering approaches

Clustering analysis is performed to infer coherent structures, often from the reduced dimensions. This involves evaluating cell-cell similarity and applying a suitable clustering algorithm to detect a certain number of segregated clusters at some resolution(s). Traditional metrics and clustering algorithms from bulk RNA-sequencing data analysis have been readily adopted in scRNA-seq analysis [105]. For example, SIMLR (Single-cell Interpretation via Multi-kernel LeaRning) utilizes Euclidean distance, Pearson’s correlation and Spearman’s correlation jointly to learn a consensus Gaussian kernel to detect diagonal block structures in these matrices [112]. Similarly, SC3 performs consensus clustering by iteratively performing PCA and k-means on a small subset of principal components, where Euclidean, Pearson, and Spearman correlations jointly evaluate the cell distances [111]. While these consensus methods over multiple similarity matrices identify robust clusters, their scalability is limited to ~ 10,000 to ~ 20,000 cells as calculation of global similarity, and consensus search are computationally expensive [111]. Density-based clustering (e.g. DBSCAN [113]) is a computationally affordable approach that searches for evenly distributed cells in lower dimension space by t-SNE or DM [9, 119]. However, these approaches may suffer stochasticity or distorted data structure due to the dimension reduction.

#### Graph-theoretic approach

Graph-theoretic approaches do not require dimension reduction and can retain both local and long-range structures in the form of cell-cell networks. k-nearest neighbor (kNN) network has been a popular method to construct these cell-cell networks, linking a cell with k most similar or closest cells [74, 92, 114–116]. In scRNA-seq settings, detection of kNN cells requires additional post-processing to account for drop-out reads and sparse expressions. The optimal partition of kNN-graph is computed through quality metrics such as Girvan-Newman (GN) modularity [120] or edge density measures. PhenoGraph constructs kNN network in Euclidean space, prunes spurious links by Jaccard index, then detects the coherent subnetworks by optimizing GN modularity with Louvain’s algorithm [114]. Louvain’s algorithm iteratively merges nodes to improve the global GN modularity, while the modularity measure acts as a scale parameter to capture from scattered and subnetworks (low modularity) to coherent and large subnetworks (large modularity) [121]. Seurat’s popular scRNA-seq analysis workflow adopts a similar strategy to PhenoGraph and allows the users to specify the resolution of resulting clusters [92]. MetaCell, on the other hand, first utilizes Spearman’s correlation in z-score transformed expression data, then applies a series of regularization steps to the adjacency matrix to remove spurious interactions, and finally identifies subnetworks with high edge densities [116]. SNN-Cliq detects dense subnetworks via quasi-clique detection in the mutual nearest neighbor network [115].

Overall, the kNN approach has become popular as it does not make assumptions about the underlying geometry. However, the choice of the kNN parameter has not reached a consensus in the field. Correlation between link weights and shared neighbors [114], global network connectivity [74] and convergence upon iterative regularizations [116] are the imporant criteria adopted by the aforementioned clustering analysis approaches.

#### Deep neural network approach

Deep neural networks consist of several layers of encoders mapping the input data into a low-dimensional manifold, from which the following decoder layers can reconstruct denoised, full-rank data. The applications in scRNA-seq include denoising single-cell transcriptome [95, 122], batch effect removal [118], probabilistic modeling of gene expressions or cell types [95, 96, 123] or dimension reduction [96, 117, 118, 124]. In cell clustering, these versatile functions of deep neural networks have become an attractive avenue to unveil complex cell architectures in scRNA-seq. Recent releases of TensorFlow [125, 126] with massive GPU parallelization have boosted the application of deep neural network learning to dissect complex patterns in in high-dimensional scRNA-seq data.

Classically, compared to the original input, these deep neural network models are trained by minimizing the reconstructed data loss. However, naïve model learning in this way could lead to over-fitting where non-biological sources of errors (e.g., drop-out reads, low coverage) in scRNA-seq contribute differently to the data noises [122]. Deep count autoencoder (DCA) and single-cell variational inference (scVI) define the reconstruction error as the log-likelihood of the noise model such as ZINB to denoise and impute the drop-out reads. The denoised data are utilized to infer cell clusters. scVI performs k-means clustering in the denoised low-dimensional latent space [96]. Similarly, scVI uses deep generative, variational autoencoder [127] with Gaussian mixture model to identify cell clusters and offer a statistically interpretable framework for downstream analyses [117]. On the contrary, DESC is a model-free approach in which a neural network generates a low-dimensional representation of the input data by minimizing the reconstruction loss [118]. An iterative clustering approach is to combine Louvain’s algorithm and cluster refinement to improve cluster purity [118].

Overall, deep neural network-based approaches offer a promising avenue to model non-linear patterns in single-cell transcriptomes, with computational scalability and flexibility to adapt different single-cell transcriptome models. However, they also face similar challenges as other approaches, such as adequate feature selections and choice of the ‘right’ models for single-cell transcriptome.

#### Genotype-based approaches

RNA reads from scRNA-seq provide a unique opportunity to infer SNVs per cell to demultiplex for individual samples [108–110], or cluster cells to trace clonal evolution [107] or genotype distributions [72]. However, challenges in scRNA-seq variant calling lurk from RNA-splicing, low transcript abundance, allelic drop-out, higher error rate from reverse transcription, incomplete transcript coverage, and 3′- or 5′-end bias in coverages [128, 129]. To handle these challenges, the pre-processing involves splice-aware alignment (e.g., STAR, minimap2), in conjunction with *mpileup* in *samtools* to detect variants present in low-coverage regions [72, 129]. To further enhance the confidence in the detected variants, pre-compiled variants from external data sets such as whole-genome sequencing (WGS) from bulk samples are used to detect the reads bearing the alternate alleles with VarTrix [72, 129].

scSplit [109], demuxlet [110] and Vireo [108] are tools dedicated to demultiplex mixed reads from individuals with known (demuxlet) or unknown genotypes (scSplit, Vireo). They are capable of detecting the doublets as outliers by the allele fraction model, which specifies the expected range of observed alternate alleles in singlet cells. On the other hand, Souporcell [72] and DENDRO [107] are specialized in clustering cells with the variant matrix to identify subclones and heterogeneity in the cell populations. Souporcell leverages mixture models to infer centroids in the alternate allele fraction space [72]. DENDRO is tuned more specifically for identifying sub-clones by measuring genetic divergence between the cells. ZINB models the allelic expressions to account for drop-out reads, and different degrees of genetic differences are utilized to construct a phylogeny tree across the cells, where each branching point characterizes sub-clonal expansion [107].

#### Evaluation of cell clustering quality

Unsupervised cell clustering is an essential component of single-cell transcriptome data analysis, and has been increasingly applied to single-cell transcriptomes [130]. However, there is no consensus on evaluating the cluster qualities to identify the best set of clusters reflecting the underlying geometry and biology in scRNA-seq. In the clustering analysis, the quality of clusters is evaluated by comparing with external gold-standard information (external validation) or the internal geometry in the data (internal validation) [131]. Internal validation evaluates intra-cluster compactness and inter-cluster separability using various indices such as Dunn’s index [132] and Davies-Bouldin index [133] that define different aspects of the underlying data geometry [134], and then determines the optimal number of clusters [134]. On the other hand, external validation evaluates how well the clusters capture relevant information outside the analyzed data. External data can be gold-standard clusters that a clustering algorithm must reproduce (e.g., known subtypes, simulated data with known clusters) [135, 136]. Their concordances can be evaluated by mutual information [137] or adjusted Rand index [134, 138].

As a rule of thumb, a good clustering analysis for scRNA-seq data in AD should reflect major cell populations with robust over-expression of the markers [26, 42, 74, 139], mix cells from different batches of samples [92], and capture key pathways associated with AD pathologies [8, 140] such as immune response, synaptic transmissions and myelination. Furthermore, the reproducibility of the identified clusters should be examined by cross-validation or bootstrapping approaches [7], concordant cell populations in animal models [140–142] or respective bulk cohorts [7, 9, 140].

#### Applications to AD

Several early scRNA-seq studies leveraged brain cells from preclinical disease models to understand cell architectures in neurodegenerative brains under controlled environments. In these studies, cell clustering analysis identified catalogs of distinct cell populations in mouse brains [119, 141], microglial subpopulations from brains undergoing neurodegeneration in mice and humans [139, 140], and differentially regulated neuronal stem cell subpopulation in AD model zebrafish [142].

Studies on the single-cell transcriptome of neurodegenerative human brains have emerged to pinpoint cell populations associated with AD-associated traits. Darmanis et al. 2015 sequenced 466 cells from healthy adult temporal lobe tissue [26]. Gaussian mixture clustering in t-SNE space revealed major brain cell types and distinct neuronal subpopulations with adult-brain-specific MHC-I expressions compared to fetal brains [26]. Olah et al. 2020 analyzed 16,242 cells from fresh prefrontal cortex samples from AD and healthy controls [7]. The study performed iterative Louvain’s clustering on different combinations of the first 15 PCs to identify robust microglial subpopulations depleted in AD [7].

The first phase of unsupervised clustering may be limited in resolution and overlook underlying fine clustering structures. Several studies biologically guided sub-clustering in major cell types to dissect distinct subpopulations. With this strategy, Lau et al. 2020 identified 43 unique cell clusters from 169,496 nuclei from prefrontal cortical samples of postmortem AD and control brains [42]. These clusters included loss of protective glial cells and enriched angiogenic endothelial cells in AD brains [42]. Similarly, Mathys et al. 2019 performed two-stage Louvain’s clustering on kNN on 80,660 nuclei from post mortem prefrontal cortices of 24 AD patients with varying pathology and 24 control subjects [8], and identified sub-clusters associated with AD-related traits and female over-representation in the AD-associated sub-clusters [8].

#### Recommended workflow: from feature selection, dimension reduction to clustering

**Fig. 3:**
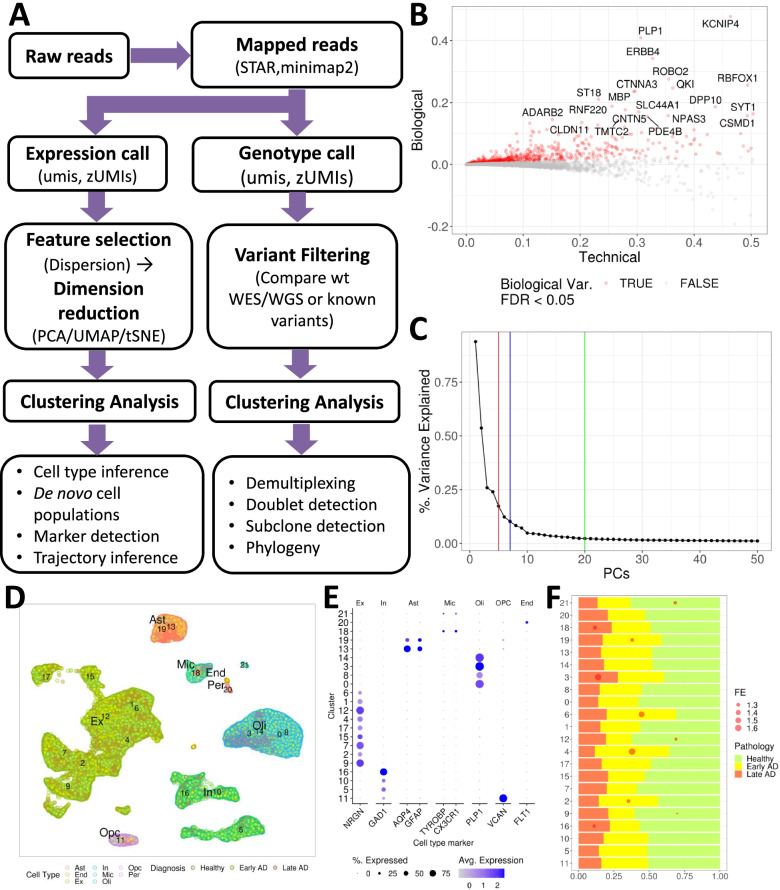
Recommended workflow of feature selection, dimension reduction, and clustering, and applications in AD. **A** Recommended workflow of dimension reduction and unsupervised clustering analysis of AD scRNA-seq data. Software tools are provided for each step. **B** Technical variance vs biological variance plot from the ROSMAP snRNA-seq data. The red dots depict genes with significantly greater biological variance than the technical variance (FDR < 0.05) and the top 20 most significant genes are labeled. **C** PC versus percentage of the variance explained. Vertical lines indicate recommended number of PCs from different workflows (red: PC denoising workflow from scran, blue: elbow point from Seurat, green: default number of PCs in Seurat). **D** UMAP plot of snRNA-seq from ROSMAP cohort. Clustering by PhenoGraph implemented in Seurat is marked by numeric labels. The cell types identified by marker gene expressions in (**E**) are highlighted as different border colors with relevant cell type name labels (Ast: astrocyte; End, endothelial; Ex: excitatory neurons; In: inhibitory neurons; Mic, microglia; Oli, oligodendrocytes; Opc: oligodendrocytes progenitor cells), and AD pathology (Healthy - green, early AD – yellow, late AD - red) are highlighted as different point colors. **E** dot plots of brain cell type markers showing their cluster-wise expressions. Clusters on the y-axis are ordered according to their proximity in the UMAP plot in (**D**). **F** Proportions of cells at different AD stages. FET is performed to evaluate whether the cells from each AD stage are enriched in each cell cluster. As significant enrichment is based on a cutoff of 0.05 for corrected FET *p*-value. In the plot, red dots represent the cases with fold enrichment (FE) > 1.3

This section illustrates the overall recommended workflow from feature selection to clustering analysis (Fig. 3A) and the scripts for these analyses can be found in the companion GitHub repository (see the section "Availability of data and software code" for details). For feature selection, gene dispersion, the gene-wise deviation from the fitted relationship between mean and variance from log-normalized expressions, can serve as the quality metrics for informative features [52] (Fig. 3A). However, in many single-cell AD studies, the cells are confounded with many ‘undesired’ variables (e.g. batches, varying sample quality, different sample preparation procedures), shadowing the meaningful biological signals, and the effects of these undesired variables should be blocked during the gene dispersion modeling [91]. We analyzed the gene dispersions in the snRNA-seq data from the ROSMAP cohort, consisting of postmortem brain tissues from 48 individuals with varying AD pathology [8] (Fig. 3B). Using scran workflow, individual-wise dispersions were first calculated, then summarized into a combined dispersion per gene. Overall, genes with significant dispersions with FDR < 0.05 exhibit high biological variances compared to the technical variances as modeled by the mean-variance curve. This is exemplified by *VCAN*, an oligodendrocyte progenitor cell marker [143], and APOE, whose polymorphism is a major genetic risk determinant of AD [144] and a marker for astrocyte and activated microglia [8] (Fig. 3B).

Then, log-normalized gene expressions across the genes with significant dispersion should be used to perform dimension reduction by PCA. Alternatively, data integration workflows (e.g. CCA [92], MNN [91] and Harmony [98]) offer adjusted features for undesired batch variables, and PCA can be applied to them. Although PCA is not a prerequisite for several down-stream analyses (e.g. deep learning-based clustering), PCA offers a time-cost effective option to identify a few key variables in high-dimensional data, and have been adopted routinely in popular scRNA-seq workflows such as scran [52], Seurat [92] and scanpy [50]. During PCA, determining the number of PCs is crucial, and several criteria such as the elbow in explained variance curve, correlations to technical variance, or PCs with significant variances when randomly permuted should be examined (see Fig. 3C). Among these criteria, random permutation-based evaluation (e.g., Jackstraw statistics in Seurat) is computationally expensive, and may not be suitable for large-scale scRNA-seq data sets (number of cells > 10,000). Instead, the simple elbow detection in the explained variance curve (blue line in Fig. 3C) can be effective without huge computational burden.

Then, the clustering analysis identifies cells with coherent expression patterns (i.e. expression-based clusters). Depending on the nature of the method, the selected gene expression features may be used directly (e.g., autoencoder-based methods), otherwise, the selected PCs should be utilized for methods relying on cell-cell distance metrics (e.g. kNN-based methods, k-means clustering). While deep learning-based methods can simultaneously handle undesired variables and capture non-linear patterns [118], they often require GPU-enabled parallel computation capacity. Thus, in the absence of such high-computation power, we recommend kNN-based methods which can capture local structures and non-linear patterns via complex network topology. Then, the selected PCs can be embedded on the lower dimensions, usually 2- or 3-dimensional space via UMAP to visualize the resulting clusters (Fig. 3D). To evaluate the clusters, the cell clusters associated with similar brain cell types such as excitatory/inhibitory neurons, astrocytes, oligodendrocytes, and microglia should express the respective cell type markers and be located in proximity as demonstrated in the ROSMAP cohort examples in Fig. 3D-E.

To further assess the biological significance of cell clusters in AD, enrichment of cells from various AD pathology (e.g. Braak staging, CERAD score, cognitive declines, AD pathology diagnosis) can guide pinpointing potential key cell populations underlying AD. We demonstrated enrichments of cells from healthy controls (no pathology), early AD (early pathology) and late AD (late pathology) from analyzing the ROSMAP snRNA-seq data (Fig. 3F) by Fisher’s Exact Test (FET). Here we used the sample pathology status defined in the original study by Mathys et al. [8]. Specifically, AD-pathology means increased AD pathological measurements such as β-amyloid (Aβ) while no-pathology represents no or very low AD pathological measurements. Based upon the degree of amyloid neurofibrillary tangle burdens, AD-pathology is further classified into two subgroups including early (modest burden) and late pathology stage AD pathology (higher burden). With a stringent threshold of 0.05 for corrected FET *p*-value < 0.05 and enrichment fold change (EFC) > 2, it readily uncovers over-represented cell populations in severe AD such as cluster 3 (an oligodendrocyte subpopulation), cluster 18 (microglial subpopulation), and cluster 16 (inhibitory neuron subpopulation).

In contrast to the expression-based clustering, the genotype-based clustering methods can facilitate several data quality control concerns when raw reads are available. For instance, cell clusters with distinct genotypes represent cells from different individuals and provide a systematic way to evaluate the agreement with the clinical annotations [145]. Further, doublet cells can be discerned via leveraging the allele fraction model (Fig. 3A).

### Cell type inference and annotation

An essential goal of clustering analysis is to characterize the identity of the cells within each cluster. Marker genes can characterize a cluster with biologically meaningful functions and inform respective cell types. For example, the cell types in the human brains can be annotated by interrogating the expression patterns of known marker genes: *NRGN* (excitatory neurons), *GAD1* (inhibitory neurons), *AQP4* (astrocytes), *MBP* (oligodendrocytes), *CSF1R* and *CD74* (microglia), *VCAN* (oligodendrocyte progenitor cells), *FLT1* (endothelial cells), and *AMBP* (pericytes) [8].

The drawback of the marker gene-based method is that the markers are often limited to major cell types, hindering the annotation of novel cell clusters or cell subclusters with unknown biological functions. To overcome this drawback, an alternative approach is to use reference signatures derived from existing single-cell datasets [146, 147]. To find the best-matched cell type, the de novo cluster marker genes can be compared with the signatures from the reference single-cell databases by enrichment test or overlapping statistics. The de novo cluster marker genes can be defined as the up-regulated genes in a cluster of interest against the rest clusters through differential expression (DE) analysis (see the Differential expression for disease gene identification section below). For large single-cell datasets, an iteration of clustering and sub-clustering analyses may be needed to reveal the structure of cell clusters. Various automated cell type annotation tools have been developed to assist with cell type annotation. For example, scQuery is a web server that predicts cell types based on over 500 different scRNA-seq experiments [148]. Garnett and scmap allow users to build their own databases or train new cell classifiers to classify cells of interest [149, 150]. These automated annotation tools can be combined with the marker gene-based methods to facilitate the annotation of large complex single-cell datasets.

### Differential expression for disease gene identification

DE analysis is useful to discover unique gene expression profiles in novel cell clusters or under disease conditions. In scRNA-seq experiments, DE analysis is presented with additional challenges such as low read depth per cell, the dropout event [151], and multimodality in gene expression values [152]. As the sequenced tissues consist of cells from different types at different states, the heterogeneity leads to variable distributions of gene expression in different cells. Moreover, the stochastic nature of transcription may introduce variability to gene expression levels [153].

A variety of DE methods have been developed to model the dropout events and the multimodal nature of scRNA-seq data. For example, MAST employs a generalized linear model (GLM) and considers the dropouts with a bimodal distribution [89]. Monocle employs a Tobit model to account for dropout events and fits the data with a generalized additive model (GAM) [89]. SCDE models the gene expression as a mixture of ZINB distributions and applies a Bayesian model to estimate the posterior probability for the DE genes [48]. D3E models gene expression distribution by the bursting model of transcriptional regulation [154]. scDD applies a multimodal Bayesian modeling framework to model the multimodal distributions of single cells [155].

To benchmark the performance of different DE methods, extensive experiments have been performed to evaluate many single-cell-based tools as well as popular bulk-tissue-based approaches. Interestingly, the comparative study showed that the single-cell-based tools did not perform better than the bulk-tissue-based methods such as limma [93], DESeq2 [156], and edgeR [157]. The performance of many tools specially designed for scRNA-seq is even worse than the simple t-test or Wilcoxon rank-sum test [158]. Both scRNA-seq and bulk RNA-seq DE tools need to strike a balance between sensitivity and precision [159, 160]. As bulk RNA-seq tools are not specifically designed to model the gene expression profiles of scRNA-seq data, they may suffer poor performance due to zero inflation or multimodality. Indeed, the performance of bulk RNA-seq tools could be further improved by combining with a weighting strategy to down-weight excess zeros [161].

#### Recommended workflow and applications to AD

Different scRNA-seq DE methods have been applied to reveal gene signatures associated with AD pathology. The bulk-tissue-based DE methods, which have efficient computational speed and sophisticated pipeline, can be directly used for the general purpose of scRNA-seq studies. For example, Grubman et al. used edgeR to identify cluster marker genes as well as the individual-specific and sex-specific differentially expressed genes (DEGs) from 13,214 nuclei of entorhinal cortex samples [41]. Meanwhile, as no single DE tool is superior in all scenarios, we recommend a combination of different methods to identify the most robust DEGs out of consensus calls. The AD study by Mathys et al. combined Wilcoxon rank-sum test and a Poisson mixed model which accounted for individual variability to identify a consensus list of 1031 DEGs in AD-pathology versus no-pathology individuals across cell types [8]. We applied MAST to the ROSMAP AD snRNA-seq data and shared the script through the companion GitHub repository (see the section "Availability of data and software code" for details).

The aforementioned DE methods depend on pre-defined cell clusters or groups, but the optimal number of cell clusters and/or biologically relevant clusters in scRNA-seq data is often hard to find out. singleCellHaystack addresses this issue by applying the Kullback–Leibler Divergence method to identify genes expressed in subsets of non-randomly positioned cells in a multidimensional space [162]. By comparing gene expression profiles to a reference distribution of all cells, singleCellHaystack can identify differentially expressed genes in an unbiased way without relying on cell clusters. The cluster-independent method may serve as a complementary approach for DE analysis when biologically meaningful clusters are not available for scRNA-seq data.

### Trajectory inference

Trajectory inference aims at estimating dynamic changes in a single-cell transcriptome landscape, assuming that the cell-wise transcriptome is a static snapshot at a time point along some cellular process. The cascades of these snapshots compose of a dynamic trajectory of cells undergoing continuous changes in the cell states, known as ‘pseudo-temporal trajectory’. Trajectory inference assigns a one-dimensional coordinate, known as pseudotime [163], per cell to approximate the departure from the beginning of the trajectory. It allows us to reconstruct dynamic biological processes without sampling tissues at different time points, identify critical transition points between distinct cell states, and analyze shifts in cell-type composition and cell synchronization [163, 164].

The inferred pseudotime may not progress uniformly in real-time along a trajectory, as the trajectory inferences are based on inferring tree-like geometry in the data rather than by ‘real world’ clocks [165]. RNA velocity provides an alternative way to time-stamp cells by utilizing RNA kinetics. According to the central dogma of molecular biology, the rate of change in mature mRNA abundance, i.e., RNA velocity, can be described by competition between mature, spliced mRNA produced from unspliced pre-mRNA and degraded mature mRNA [166]. In this framework, a greater abundance of pre-mRNA than the mature mRNA indicates an up-regulation, and a down-regulation in the contrary [166, 167]. The summarized kinetics in the global cell transcriptome can facilitate trajectory inference [168].

Information on cellular dynamics could improve our understanding of AD pathologies, such as identification of marker genes for early diagnosis and prompt intervention of neurodegenerative diseases whose pathogenesis precedes many years before clinical manifestation. Herein, we review different computational approaches in cell trajectory inference and discuss its outlooks in AD scRNA-seq analysis.

#### Overview of trajectory inference methods

Inspired by the metaphorical epigenetic landscape conceived by Waddington, Trapnell et al. adopted a dynamical systems framework. They described the biological process as cells moving in the “gene regulation space” along a particular “trajectory” to finally reach a stable state that corresponds to a clearly defined cell type or an “attractor” in dynamical systems [169]. In this framework, trajectory inference consists of three components: determination of gene regulation space (dimensionality reduction), identification of the attractors (unsupervised cell clustering), and the inference of the trajectory (graph-based data approximation followed by pathfinding and cell ordering). Here, we will primarily focus on the third component as the first two have been extensively discussed in the prior sections in this review.

Graph-based data approximation is used to extract the geometrical skeletons of a given data point cloud. Such graph types include, for example, principle curves [170], minimum spanning trees (MST) [171], nearest neighbor (NN) graphs [172], and more complex networks. Early trajectory inference methods contemplate the trajectory structures to be non-branching (Wanderlust [173]), bifurcated (Wishbone [174]), or even cyclic (DeepCycle on single-cell imaging data [175]), and require prior biological knowledge or user-provided input. Emerging methods, some of which will be covered subsequently, allow unbiased inference of trajectory structures from transcriptomic data at the cost of increased computational complexity, which would impact their scalability and usability.

MST is a tree-graph which spans the entire data points with the minimum overall distance. While each node in the MST represents a single cell, the edge can be the similarity between gene expression profiles or transition probability between neighboring cells. Monocle, one of the pioneer algorithms for trajectory inference, applies the independent component analysis (ICA) and constructs an MST over all the cells [163]. SCOUP models the probability of a cell differentiating into a neighboring cell in a PCA-reduced space based on the Ornestain-Uhlenbeck (OU) process and assumes that a mixture of OU processes represents multiple cell fates during differentiation defined by the shortest paths in the MST [176].

As MST is often sensitive to noise and outliers, Waterfall [177] and TSCAN [178] construct a cluster-based MST to improve the robustness. Slingshot takes one step further by implementing simultaneous principal curves compatible with any dimensionality reduction method to infer multiple fates that individual cells may take during development [106].

Some algorithms use the kNN graphs to overcome the impact of noise and outliers. SLICER takes the shorted path on a kNN graph in a reduced space by locally-linear-embedding (LLE) and determines the branching location by geodesic entropy [179]. Diffusion Pseudotime (DPT) takes a random walk in the nearest neighbor graph in the high-dimensional space. The pseudotime is inferred by Euclidean distance between the probability vectors, rather than gene expression, of any two cells differentiating into all possible fates [102]. However, it might be inappropriate to use a fixed neighborhood size in some cases, as the data are not evenly distributed across the defined space. Moreover, the computational cost of kNN increases drastically with the number of cells.

Others construct complex networks for cell projection to allow assumption-free inference of trajectory topologies. For instance, scEpath [180] builds an energy landscape and infer transition probabilities and lineage relationships between cell stages. Hopland [181] maps cells onto Waddington’s epigenetic landscape and infer pseudotime sequences by geodesic distance.

Another attention-drawing question is whether a continuous transition process is presumed in the trajectory inference algorithms. While the answer is yes in most cases, some would argue that due to limited sampling rate/depth, the experimental data do not always conform to such assumptions. Several methods have been developed to tackle this issue. For example, PArtition-based Graph Abstraction (PAGA) [182] models the connectivity of cell groups and reconstructs both continuous and disconnected topologies at multiple resolutions [183]. Monocle 3 [184] adopts a similar idea to PAGA. It first projects the cells onto a lower-dimensional manifold by UMAP and merged adjacent groups of cells identified by the Louvain community detection algorithm into “supergroups” to resolve the developmental trajectories. Another example is TinGa, a growing neural graph-based algorithm that also allows disconnected topologies [185].

#### Overview of RNA velocity

The balance between spliced and unspliced mRNA, termed RNA velocity, measures the transcriptional dynamics in the cells and facilitates trajectory inference. In scRNA-seq, Manno et al. 2018 first utilized the relative abundances of exonic and intronic reads to infer the cell-level RNA velocity with a simplified model assuming the same rate of pre-mRNA processing for all genes [166–168, 186, 187]. The cell-level RNA velocity inference was applied to scRNA-seq data of mammalian embryo brains and captured dynamic changes in developmental trajectories [168, 187]. Bergen et al. 2020 developed scVelo to implement a more generalized kinetic model with gene-specific pre-mRNA processing rate and infer the kinetics-based cell trajectories in scRNA-seq [187]. While RNA velocity was analyzed mostly in developmental processes, these have not been applied in AD single-cell transcriptome. Potentially, RNA velocity underlying AD-specific microglial or neuronal subpopulations may shed light on key dynamical splicing activities contributing to these AD-specific cell fates.

#### Recommended workflow and application of trajectory inference to AD

**Fig. 4:**
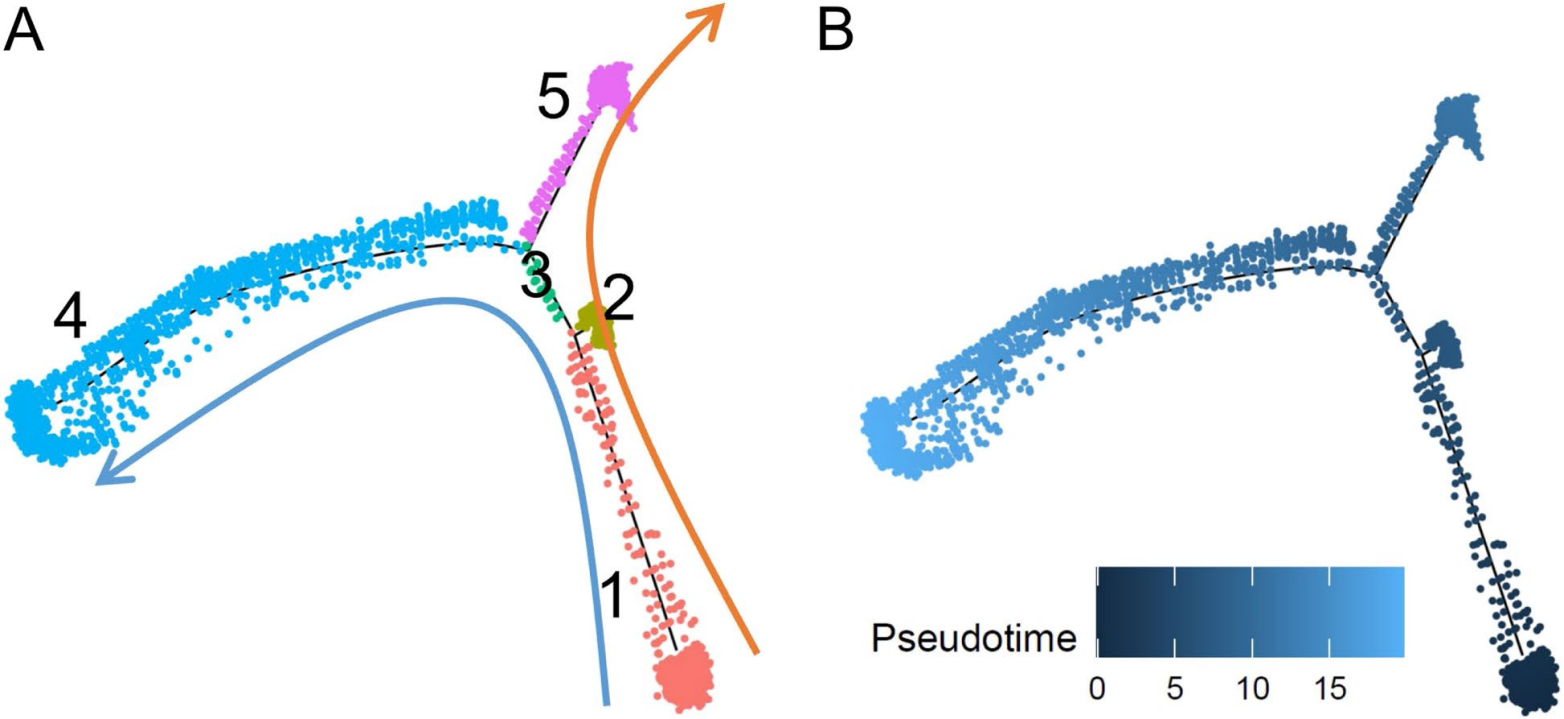
Trajectory inference of an excitatory neuron cluster of the ROSMAP AD snRNA-seq data. **A** Numbered cell states in different colors and two potential trajectory paths as indicated by curved arrows. **B** Pseudotime indicated by gradient color intensity

A typical workflow may involve the following steps: 1) conduct data QC, normalization, dimension reduction, and clustering as described above or according to the trajectory inference software package; 2) choose genes that are informative of the cell state progress, such as cell type markers and highly variable genes; 3) conduct data dimension reduction; 4) infer the trajectory and order cells by pseudotime in the trajectory; 5) identify genes regulated over the course of trajectory, such as genes that correlate with the pseudotime or distinguish between cell state along the trajectory branches; and 6) perform additional analyses, such as constructing Granger’s causality network using pseudotime information and identifying trajectory path that correlate with covariates of interest such as AD pathology traits; and 7) generate biologically meaningful hypothesis for experimental validation. As an example, we conducted a trajectory inference for an excitatory neuron cluster of the ROSMAP AD snRNA-seq data using Monocle (Fig. 4). A pipeline implementing this trajectory analysis is provided in the companion GitHub repository (see the section "Availability of data and software code" for details).

scRNA-seq-based trajectory inference methods have been extensively utilized to study the developmental processes and immune systems where cells undergo active transitions from one state to another [188–192]. A unique disease-associated microglia subtype was identified in AD transgenic mouse brains by trajectory inference [139]. Several AD cohort studies [193–195] generalized this concept and aligned the individual subjects along the disease trajectory. The inferred models successfully predicted the clinical deterioration and conversion to advanced disease stage from baseline gene expression and disease subtype stratification.

With the development of high-throughput single-cell sequencing techniques, multi-omics data can be simultaneously measured in the same cell. G&Tseq sequences both genome and transcriptome [196], REAPseq measures protein and transcripts [197], scTrio-seq [198] quantifies genetic, epigenetic, and transcriptomic changes, and paired-seq [199] jointly examines the chromatin accessibility with transcriptomic heterogeneity. Despite the modest coverage, such methods add comprehensive information to help infer the trajectory of cells. Algorithms have been developed to compare cross-experiment, cell-type-specific differences and integrate multi-omics at the single-cell scale [200]. Recently, a microarray-based spatial scRNA-seq further resolves the spatial distribution of cell subpopulations in pancreatic tumors [201]. Such progress will prime the ground for novel findings in complex diseases, including AD.

Trajectory inference opens new venues to capture biologically critical dynamic changes that were considered as noises, and enables additional insights via trajectory-based differential analysis [202–204], latent variables-pseudotime interactions [205], pseudotime-based gene co-expression network analysis [206], and gene regulatory network inference [207], which will be discussed in the section “Single cell gene network analysis”.

### Copy number variation detection

While scRNA-seq is primarily designed to quantify the cell-level expression abundance, the sequencing data contains a substantial portion of the information about the genomic variations, including SNPs and Copy number variations (CNVs). CNVs are one major type of genetic variation. Based on the CNV map of the human genome [208], CNVs occupy 4.8 to 9.5% of the human genome in healthy individuals [208–210]. Copy number aberration is involved in the pathogenic process of many diseases, for example, a variety of cancers [9, 211–214], Parkinson’s disease [215, 216], schizophrenia [217], mental retardation [218], and AD [218–222]. Our recent study found thousands of AD-specific CNVs based on bulk-tissue based whole-genome sequencing data of postmortem brains from AD cases. Whether there are cell- or cell cluster-specific de novo CNVs in AD remains unclear.

Compared with many previous successful CNV calling methods based on bulk tissue sequencing data [223–233], CNV detection from scRNA-seq is challenging due to several technical limitations, including low and non-uniform genome coverage, amplification biases [234, 235] and prevalent monoallelic detection due to transcriptional stochasticity [234, 236–238]. The monoallelic bias is more pronounced for lowly expressed genes than highly expressed genes. The monoallelic bias is still high for polymorphic loci with good coverage [237, 238]. Further, 3′-ended scRNA-seq have poor coverage in the 5′-end. Thus, the mutations in the 5′ end may not be sufficiently covered. Together, these limitations reduce the reliability of CNV calling at the gene level in scRNA-seq. Instead, previous studies have suggested large-scale CNVs can be reliably inferred from scRNA-seq at full chromosome-level or chromosome-arm-level [212, 239, 240].

Despite these challenges, several methods, including InferCNV [241], HoneyBADGER [236], CONICS [239], CONICSmat [239], and CaSpER [242] have been developed to detect CNVs from scRNA-seq data (Table 3). InferCNV [241], as a part of the TrinityCTAT toolkit, is the first and the most popular scRNA-seq CNV detection method to predict chromosome-scale CNVs. It calculates residual transcriptomic expression profiles of target cells using a given set of normal reference cells as the baseline. It identifies potential copy number alteration (CNA) regions using the Hidden Markov Model (HMM). To reduce the false-positive rate, a Bayesian mixture model is further implemented to estimate the copy number status of each CNA region in each cell based on the maximized posterior probability [241]. It can work with and without normal-cell reference. If there are no reference cells, the average signal of all target cells will be used as the baseline. It should be kept in mind, though, CNVs shared by all target cells are indistinguishable without reference cells [241]. HoneyBADGER [236] was developed based on a similar algorithm framework as InferCNV, which integrates the HMM and Bayesian inference. To improve the CNV calling accuracy and sensitivity, HoneyBADGER takes continuous allelic imbalance patterns at common SNP loci into consideration. The monoallelic bias rate is also adjusted in their posterior probability model. Based on in-silico simulation, HoneyBADGER can identify sub-clonal CNVs as small as 10 Mb, and chromosome-arm-level CNV events with cell frequency as low as 30%. The inference resolution of the method is higher than the solely normalized expression profile-based method [236]. Like InferCNV, HoneyBADGER also needs expression profiles of normal cells as a reference to calculate residual expression magnitude. Besides the normal cell reference, it also needs whole-genome sequencing or whole-exome sequencing (WES) data from the same sample to get heterozygous SNP positions. If WES data from the same sample is not available, the common SNPs (population frequency ≥ 10%) from natural populations can be used as a location reference. Furthermore, another scRNA-seq CNV calling method CONICS (**CO**py-**N**umber analysis **I**n single-**C**ell RNA-**S**equencing), released in 2018 [239], infers the CNV status of given cells based on pre-inferred CNV regions from additional bulk-tissue DNA sequencing data (for example, WES data). If the extra DNA-sequencing data or control scRNA-seq data is not available, it also provides an extra caller named CONICSmat. CONICSmat is based on Bayesian inference from averaged gene expression profiling of target scRNA-seq data. HMM is not used to infer potential CNV region in CONICS/CONICSmat. Potential CNV location is inferred from additional bulk-tissue DNA sequencing data. As a result, its resolution depends on the resolution of the bulk-tissue DNA CNV calling method. Without extra bulk-tissue DNA data, the CNV inference is chromosome-arm level [239]. CaSpER [242], which adopted a strategy very similar to HoneyBADGER, integrates allele frequency shift information and normalized expression profiles to predict CNV regions using hierarchical HMM and Bayesian algorithms. It does not need prior variant calling. To speed up the whole CNV calling process, CaSpER takes aligned bam files as input to generate allele and expression profiles.

**Table 3:** Summary of CNV calling methods for scRNA-seq data

Method	Brief Explanation	Input	Resolution	Advantages	Disadvantage
**InferCNV**	Hidden Markov model: i3 and i6 model + Bayesian analysis. The i3 model: deletion, neutral and amplification states. The i6 model: complete loss, loss of one copy, neutral, addition of one copy, addition of two copies, and more than three copies.	Expression profiling	Identification of large-scale chromosome-scale CNVs	1) InferCNV can work both with and without normal-cell reference; 2) it provides two analysis modes including predefined cell types as whole samples, or subclusters based on CNV patterns; 3) InferCNV provides an interactive R Shiny Web App	InferCNV assumes the copy number dosage is constant over the whole predicted region.
**HoneyBADGER**	Hidden Markov model and Bayesian approach	Allelic imbalance and normalized expression profiling	Robust identification of sub-clonal focal alterations as small as 10 Mb; identification of CNVs at chromosome-arm-level with frequency as low as 30% of target cells, and at the full chromosome-level.	1) Identifcation of CNVs as small as 10 Mb, much higher compared with average expression-based methods; 2) Detection of detect copy-number neutral loss-of-heterozygosity events.	1) Use of WES or common natural SNP information from other public datasets as reference to generate heterozygous SNP positions; 2) Instead of estimating precise copy number, it aims at distinguishing copy number alteration regions from copy number neutral regions.
**CONICS**	Comparison of control distribution and observed distribution at each CNVR region in each cell.	Expression profiling	CNV regions inferred from other DNA sequencing data or the chromosome-arm level.	CONICS provides routines for further differential-expression, phylogeny, and co-expression network analysis.	1) Predefined CNV locations in orthogonal DNA sequencing data such as WES. 2) Incapable of identifying novel CNV regions.
**CONICSmat**	Bayesian approach: chi-squared likelihood-ratio test by comparing 2-component Gaussian mixture model and 1-component Gaussian model.	Expression profiling	chromosomal-arm-level	1) No need of an explicit normal control dataset, or DNA-sequencing data; 2) Providing routines for further differential-expression, phylogeny, and co-expression network analyses.	1) Identification of CNVs at the mega base scale. 2) Incapable of identifying gene-level CNVs.
**CaSpER**	Hidden Markov model and Bayesian approach	Allele frequency shift+ expression profiling	large-scale gene-based, and segment-based CNV calls	1) Variant calling is not needed and this can speed up the whole detection process; 2) CaSpER provides a number of downstream analyses: infer clonal evolution, discover mutual-exclusive and co-occurring CNV events, identify gene expression signature of the identified clones.	1) The true positive rate only reaches 60–80%. 2) The detection accuracy for deletion is much higher than amplification.

Here, we present the pros and cons of all methods, with consideration for budget and experiment design (Table 3). While methods utilizing both allelic-imbalance and gene expressions could improve the accuracy and sensitivity of CNV calls, the additional DNA-sequencing data required to generate allelic-imbalance profiles need additional budgets and sample materials. For example, some tools such as HoneyBADGER bypass such issues by leveraging common SNPs from public population datasets instead of the matched WES data in exchange for reduced sensitivity. On the other hand, the performance of expression-based methods is dependent on reference cells of choice [236]. Ideally, matched normal cells from the same individual could be used as references, or germline cell populations may serve as the alternative [9].

An independent normalization procedure of diverse cell types should be done based on the corresponding references [236]. All scRNA-seq CNV methods are highly coverage-dependent [236]. The true positive rate (TPR) of scRNA-seq CNV calling methods is not high in the current stage. For example, the TPR of CaSpER is around 60 to 80% based on in-silico simulation [242]. Additional experiment methods should be used as cross-validation [9], such as fluorescence in situ hybridization (FISH), cytogenetics and bulk WES or WGS. Matched scDNA-seq and scRNA-seq data [196, 243] can be used as ground truth to measure the performances of current scRNA-seq CNV tools, and be valuable for future software development.

All the above scRNA-seq CNV calling tools provide some downstream analysis, such as cell clustering [241], inter-clone differential expression analysis [239], phylogeny analysis [239], intra-clone co-expression network analysis [239], infer clonal evolution [242], and identify gene expression signature of clones [242].

#### Recommended workflow and applications to AD: copy number variation detection

**Fig. 5:**
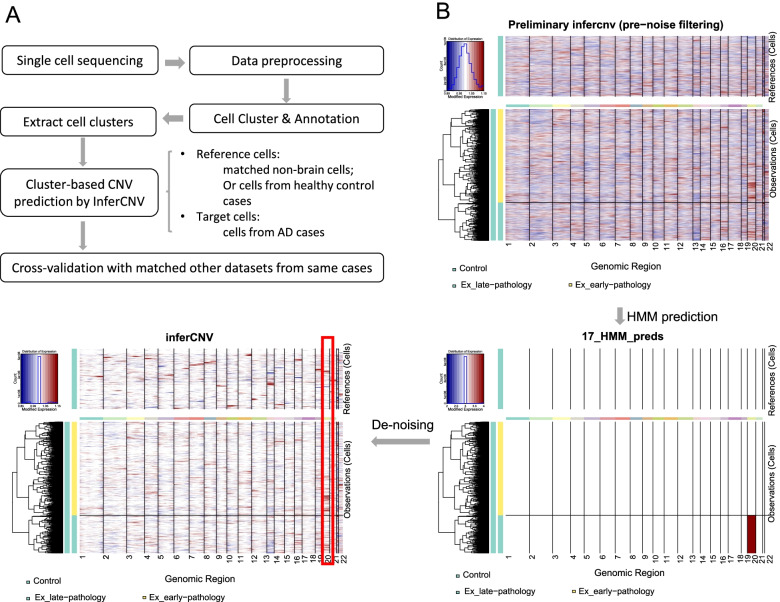
CNV identification from AD single cell data. **A** Recommended workflow of CNV detection. **B** Inference of CNVs in excitatory neurons of the ROSMAP cohort. The red box highlights an amplification region on chromosome 19

A recommended workflow for identifying CNVs in single cell RNA-seq data based on the InferCNV tool is illustrated in Fig. 5A. Note that the data preprocessing step will follow prior discussions in Quality control and normalization, Feature selection and dimension reduction, Unsupervised cell clustering analysis and Cell type inference and annotation sections. There can be two kinds of reference cells in AD. First, for each cell cluster, cells from normal controls can be used as the reference cells and can be compared with cells from AD cases in the same cell cluster. Second, brain tissue-based cells can be compared with matched non-brain tissue cells from the same individual, for example, blood, to detect brain-specific somatic CNVs. Further omics data, such as genomic data, are needed to validate the inferred CNVs in both cases.

We applied this workflow to the sn-RNAseq data from the ROSMAP AD cohort [8]. Figure 5B shows the CNV calling result from the AD cells in one excitatory neuron cluster when using the cells from normal controls in the same cluster as the reference cells. The only predicted CNV region is located at chr19: 571,277-55,403,250 with an extra copy in late AD (Supplementary Table S1). This amplification region contains 66 genes (Supplementary Table S2), including *PPP2R1A*. *PPP2R1A* is known AD risk factor [244] and dephosphorylates tau protein [245, 246]. The script for this CNV analysis can be found in the companion GitHub repository (see the section "Availability of data and software code" for details).

### Expression associated quantitative trait locus (eQTL) analysis

Expression quantitative trait loci (eQTLs) analysis links single nucleotide polymorphisms (SNPs) with their potential transcriptional effects on downstream genes [247] and has been utilized to pinpoint disease-risk SNPs [248]. Previous studies have shown that disease-risk SNPs are enriched for cis-eQTLs with modest effects [249, 250]. Many large-scale eQTL consortiums have emerged in recent years, such as ImmVar [251], BLUEPRINT [252], GTEx [253], CAGE [254], PsychENCODE [255], and eQTLGen [256]. Although bulk-tissue-based eQTL analysis is still valuable to understand the functional consequences of genetic variations, it has limited power to decipher the context-specific eQTLs, such as the tissue-specificity [247, 253, 257], cell type-specificity [247, 248, 258–260], and developmental stage-specificity [251, 261]. These are further complicated by transient eQTLs and those conditional on cell status [247, 261]. Single-cell eQTLs (sc-eQTLs) analysis can shed light on these issues.

#### Detection of eQTLs from scRNA-seq data

In sc-eQTL analysis, the number of cells should be large enough for sufficient statistical power [247]. At least three parameters need to be considered in the experimental design stage: the sequencing depth per cell, the number of cells per individual, and the number of individuals. The sequencing depth per cell affects the accuracy of gene expression measurement, the total number of cells affects the cell type number, and the number of individuals affects the effective SNP number [247]. Under a fixed budget, sequencing more cells rather than sequencing more reads per cell in fewer cells can increase the power to detect more sc-eQTLs [247].

The sparsity of scRNA-seq makes it less powerful to detect sc-eQTLs than bulk-tissue data [247, 262]. Although the analytic procedure is straightforward, scRNA-seq data may not meet the underlying assumptions for bulk tissue-eQTL methods [247]. For instance, bulk-based methods assume that log-transformed gene expressions follow a particular probability distribution (normal distribution, Poisson distribution, or negative binomial distribution), which may not be valid in scRNA-seq [247]. Further, drop-out events introduce a bias towards highly expressed transcripts [262], and the sparse transcriptome decreases the number of genes with detectable eQTLs [247, 261–264]. Previous studies show a 6.9-fold difference in the eQTL detection power between single-cell data and bulk RNA-seq data [247, 249, 260].

Several softwares have been developed to address the above challenges in sc-eQTL detection, such as SCeQTL [262] and scReQTL [265]. While dropout reads can be imputed to mitigate the sparsity [81–83, 260], SCeQTL (**S**ingle **C**ell **e**xpression **Q**uantitative **T**rait **L**ocus) [262] incorporates the excess of zero expressed genes into the statistic inference framework. SCeQTL separates genes with zero and non-zero expression, and uses zero-inflated negative binomial regression [266].

In addition to sparsity in scRNA-seq, other factors like cell lineages and variant allele frequency can also be incorporated into the inference framework [262]. Inferred single-cell pseudo-time can be utilized to capture eQTLs related to cell differentiation [261]. scReQTL [265] calculates the correlation between variant allele fraction at biallelic polymorphism loci (VAF_RNA_) and gene expression level in single cells, using a linear regression model. VAF_RNA_ is derived from allele mapping of scRNA-seq data and is sensitive to allele mapping bias. SNP-aware alignment is preferred in the preprocessing step [265]. Prevalent monoallelic expression and single-cell sequencing technique bias towards 3′-end (for example, 10X Genome platform [37]) limit the detection power of scReQTL. scReQTL can only detect a subset of expressed SNPs from the genome-wide SNP profiles. This approach is suitable for single-cell data without matched DNA sequencing information.

Single-cell-based eQTL analysis is still in its infancy. Even though the proof of concept started in 2013 [267], the real application of sc-eQTL analysis started just recently [110, 260, 261, 268]. As one major type of molecular marker like CNV, sc-eQTLs can be used to infer cell type [247, 260], study cell-to-cell expression variability [261], cell type heterogeneity [269], and cell lineage development [247, 260]. It has been shown that the heritability of diseases and complex traits can be explained partially by cell type-specific eQTLs [248]. Other unique advantages of sc-eQTLs are inferring the cell activation states [247, 260] and studying the dynamic process of genetic variations regulating gene expression [260, 261, 268]. Integration of sc-eQTLs and other omics data, for example, scATAC-seq data, can help better understand the genetic mechanism of gene expression regulation at cell type level [247, 261]. Matched scDNA-seq and scRNA-seq datasets can provide higher resolution in sc-eQTL analysis. Unfortunately, current public datasets with paralleled scDNA-seq and scRNA-seq are still rare [262]. Single-cell eQTLGen consortium (sc-eQTLGen) spearheaded the efforts to link disease-related genetic variations with downstream transcriptional consequences in immune cells [147].

#### Recommended workflow and applications to AD: eQTL detection

**Fig. 6:**
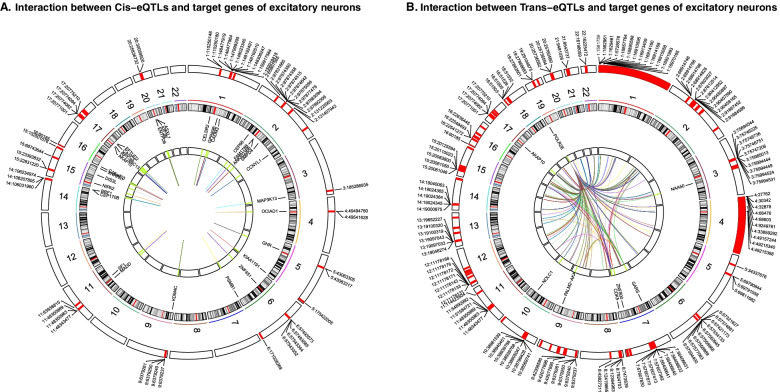
Single cell based eQTL detected in the excitatory neurons in the AD cases of the ROSMAP cohort. The outer track shows eQTL locations. Red bars indicate eQTL locations. The middle track shows chromosome ideogram. The inner track shows correlated genes. The colorful lines in the inner circle link the eQTLs to the target genes. **A,** cis-eQTLs. **B**, trans-eQTLs

We recommend inferring sc-eQTL by summarizing gene expressions by distinct cell populations [247, 260, 261]. The normalized gene expression matrix is averaged per gene, cell type, and individual to derive robust expression values per group to overcome cell-wise sparsity. The summarized gene expression is further integrated with the genotype matrix to identify sc-eQTLs via Spearman rank correlation [270, 271] or linear regression [260, 261, 272, 273]. As an example, we applied this workflow in the ROSMAP AD snRNA-seq cohort and identified cis- and trans-eQTLs in the excitatory neurons of AD cases (Fig. 6). The script for this eQTL analysis can be found in the companion GitHub repository (see the section "Availability of data and software code" for details).

### Single-cell ATAC-seq data analysis

**Fig. 7:**
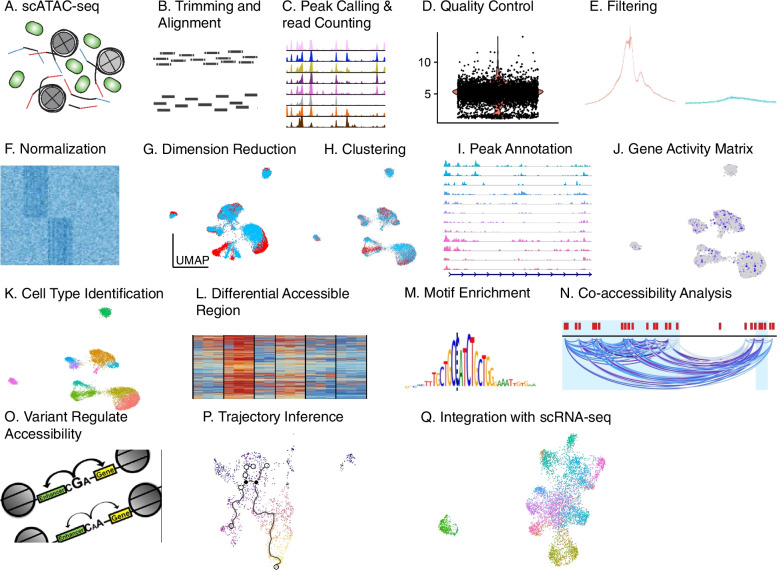
Overview of the procedures for analyzing scATAC-seq data. scATAC-seq raw data are collected from sequencing machines (**A**). Sequencing adaptors are trimmed and reads are then aligned to the reference genome (**B**). Peaks are called for each cell and merged into a set of unique features (peaks); reads are then counted for each feature in each cell to obtain a feature-by-cell matrix (**C**). Features and cells go through quality control (**D**) to remove low-quality features and cells (**E**). Filtered data are then normalized (**F**). Top variable features are extracted to perform linear and non-linear dimension reduction (**G**) that are further utilized for clustering analysis to identify cell clusters (**H**). Features (peaks) are annotated to gene (**I**) and reads are counted for each annotated gene in each cell to obtain a gene activity matrix (**J**). Cell-cluster-specific accessible chromatin regions and cell-cluster-specific activated genes are identified for each cell cluster to identify cell type (**K**). Genes with differential activity and differentially accessible chromatin regions are identified between conditions in each cell type (**L**). Transcription factor motifs are identified in each cell type (**M**). Co-accessibility analysis can be performed to infer cell-type-specific interactions between different genomic elements (**N**). Function of disease-associated genetic variant can be inferred by integrating them with scATAC-seq data (**O**). Trajectory analysis can be performed to infer cellular dynamics during developmental or disease progression (**P**). When available, scRNA-seq can be integrated with scATAC-seq data (**Q**) to infer *cis*-regulatory network

Although scRNA-seq improves our ability to study gene expression variations and interactions among different cell types in the brain, the fundamental mechanisms that regulate the variability with chromatin structure variations remain unclear. The scATAC-seq (Fig. 7A) technology has been developed to study these regulatory elements [20, 21]. Compared to scRNA-seq data, the scATAC-seq feature matrix data is sparser and hence more challenging to analyze [21, 274]. Many computational tools (Table 4) have been developed to analyze scATAC-seq data alone [275–277] or integrate scATAC-seq with other single-cell omics data, including scRNA-seq [278, 279], protein profiling [280] and genome variants [281].

**Table 4:** Summary of scATAC-seq analysis tools

*Tool [Ref]*	*Feature Matrix*	*QC*	*Clustering*	*Gene activity*	*DAR*	*Motif*	*scRNA-seq integration*	*Platform*
Signac [282]	Peak	YES	YES	YES	YES	NO	Seurat	R
ArchR [283]	Peak/Bin	YES	YES	YES	YES	NO	Seurat	R
chromVAR [275]	TF motifs	YES	YES	NO	NO	YES	NO	R
SnapATAC [284]	Peak/Bin	YES	YES	YES	YES	YES	Seurat	R/Python
cisTopic [277]	Peak	YES	YES	YES	NO	NO	NO	R
SHARE-seq [278]	Peak	YES	YES	YES	NO	YES	Seurat	R
BROCKMAN [285]	Peak	YES	YES	NO	NO	YES	NO	R
AtacWorks [286]	Peak	YES	NO	YES	YES	NO	NO	Python
Destin [287]	Peak	YES	YES	NO	YES	NO	NO	R
EpiScanpy [288]	Peak	YES	YES	NO	NO	NO	NO	Python
Cicero [276]	TSS	YES	YES	YES	YES	NO	NO	R
Scasat [289]	Peak	YES	YES	NO	YES	NO	NO	R/Python
SCRAT [290]	Any	YES	YES	NO	YES	NO	NO	R
SCALE [291]	Peak	YES	YES	NO	YES	YES	NO	Python
ChromSCape [292]	Peak/Bin/TSS	YES	YES	NO	YES	NO	NO	R
Cusanovich2018 [293]	Peak	YES	YES	YES	YES	YES	Seurat	R
scABC [294]	Peak	YES	YES	NO	NO	YES	NO	R
scATAC-pro [295]	Peak	YES	YES	YES	YES	YES	NO	R/Python

*QC* Quality Control, *DAR* Differentially accessible region

The preprocessing steps of scATAC-seq data analysis include data demultiplexing if multiple samples are sequenced simultaneously. This is followed by adaptor trimming, alignment to the reference genome [21, 296], peak calling and merging, read counting, QC, data normalization and transformation, dimension reduction, clustering, and cell identity annotation (Fig. 7B-K). Generally, peaks are called using the MACS2 [297] and then merged to generate a list of potential regulatory elements, termed features herein for simplicity. Reads of each cell are then counted for those features to obtain a feature-by-cell matrix (Fig. 7C). Next, QC is performed on the cells and features to remove low-quality cells and features [282] (Fig. 7D). The filtered feature-by-cell matrix (Fig. 7E) is usually normalized using the term-frequency inverse-document-frequency (TF-IDF) method to normalize the matrix across cells to correct for differences in sequencing depth and give more weight to rare and more variable peaks [277, 293, 294] (Fig. 7F). To reduce redundant information, potential noise and computational time for downstream analysis, dimension reduction is performed after selecting the features (Fig. 7G). Typically, Latent Semantic Indexing (LSI) is applied on the TF-IDF normalized matrix, followed by singular value decomposition (SVD) [282, 293]. Alternative dimension reduction methods include Multi-dimensional scaling (MDS) [289], Diffusion map (DM) [284], and Latent Dirichlet allocation (LDA) [277]. After feature selection and dimension reduction, samples from multiple conditions are integrated, and adjusted for batch effect [282, 293]. Then, non-linear dimension reduction approaches like t-SNE [298] and UMAP [103] are performed to visualize cells in a 2-D or 3-D space. Cell clustering is then performed in the reduced dimensions (Fig. 7H).

After clustering analysis, annotating cell identity for each cluster is a critical step. As lack of cell-type-specific chromatin accessibility features, peaks at promoters and transcription start sites (TSSs) are used in cell cluster annotation by taking advantage of the extensive cell-type-specific genes. For this purpose, peaks associated with regulatory regions and genic regions are annotated (Fig. 7I). Then, a gene activity matrix is created from the scATAC-seq data by summing the reads intersecting peaks associated with regulatory regions and genic regions for each annotated gene (Fig. 7J). Based on the gene activity matrix, two strategies are used to annotate the cell identity of the clusters (Fig. 7K). In the first strategy, gene activity markers are identified and compared to cell-type-specific marker genes [293]. In the second strategy, scATAC-seq gene activity matrix can be projected to the matched scRNA-seq gene expression matrix for the same cell types. Cell type labels can be transferred from the scRNA-seq data to the scATAC-seq data using mutual nearest neighbors (MNN) algorithm [92, 282, 293].

Then, chromatin accessibility can be investigated for individual cell types with or without integrating other omics data [21, 92, 278]. For example, brain regional and cell-type-specific chromatin accessibility dynamics in AD can elucidate the chromatin regulation mechanisms of gene expression changes underlying AD etiology (Fig. 7L). Specifically, such analyses can identify cell-type-specific peaks, differentially accessible regions [282, 299], enriched motifs (Fig. 7M) and co-accessibility [276, 299] (Fig. 7N), and infer cell trajectory [276, 300] (Fig. 7P). In addition, changes in transcription factor (TF) activity can be inferred through calculating the correlations between TF motif-related chromatin accessibility [275] and gene expression levels of AD-associated genes. Furthermore, the function of disease-associated genetic variants identified in the genome-wide association studies (GWAS) studies can be inferred by predicting genetic variants in chromatin accessible peaks that affect regulatory interactions and TF binding (Fig. 7O). For example, Corces et al. identified the SNPs that drive the association of AD with *BIN1*, *PICALM*, *SLC24A4,* and *MS4A6A* in specific brain cell types using the scATAC-seq data and AD-associated SNPs [281]. Finally, *cis*-regulatory network can be inferred by integrating scATAC-seq and sc-RNA-seq data (Fig. 7Q).

**Fig. 8:**
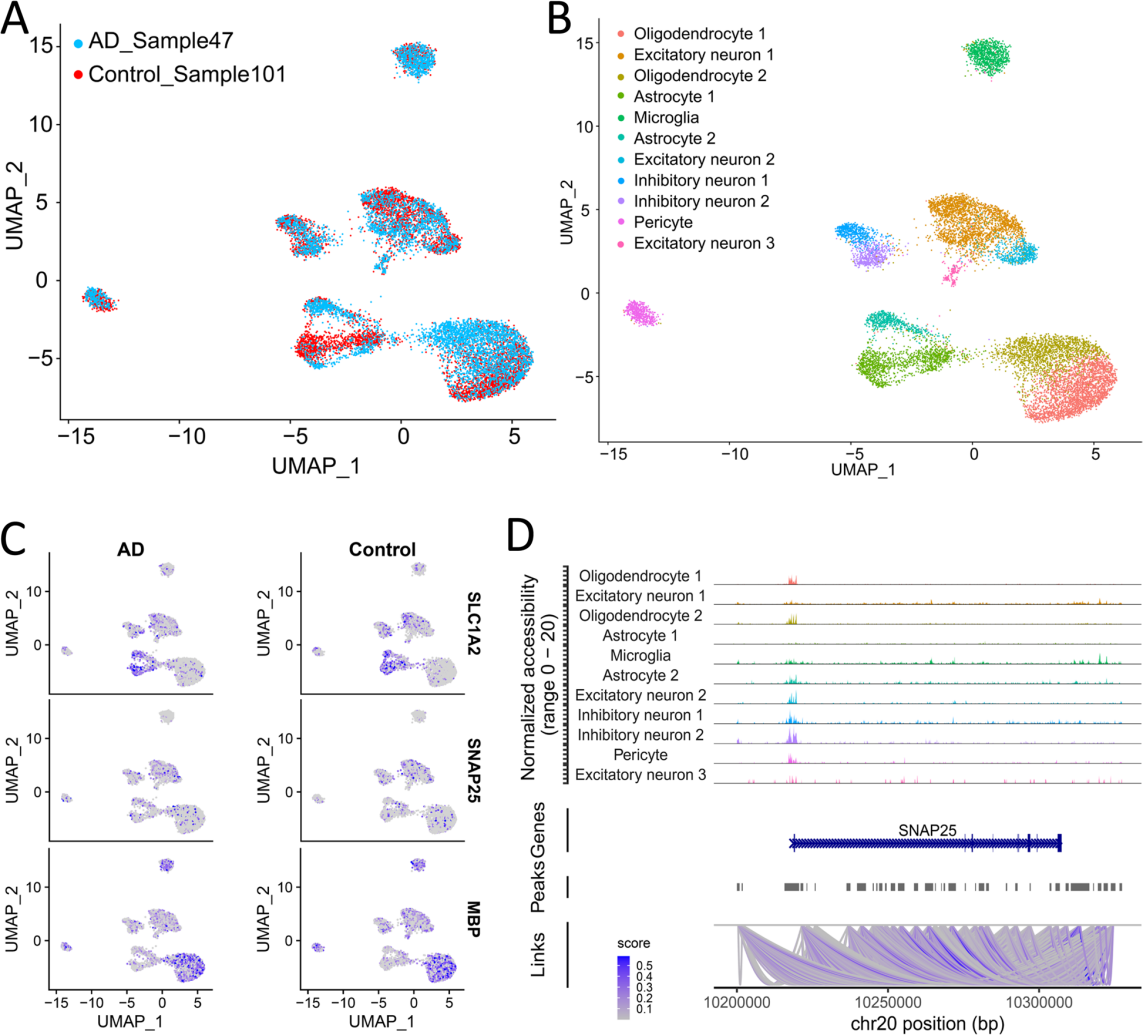
snATAC-Seq analysis for AD and control. **A** Sample 47 (AD) and sample 101 (Control) from the Swarup study are integrated and projected onto UMAP. **B** Cells are clustered into 11 cell clusters. Gene activity matrix is extracted from fragment file. Marker genes are then identified for each cluster and cell types are identified according to the marker genes of each cluster. **C** Gene activity of *SLC1A2*, *SNAP25* and *MBP* are shown in AD (left) and control (right), respectively. **D** Accessibility and co-accessibility of *SNAP25*. The top panel shows normalized accessibility of 11 cell types. The two panels in the middle show the peaks around *SNAP25*. The bottom panel shows the co-accessibility scores among the peaks

We applied the Signac pipeline to snATAC-Seq samples (an AD and a control) from the Swarup lab [PMID: 34239132]. The two samples were normalized and integrated into a dataset (Fig. 8A). Gene activities were extracted from fragments and then 11 cell types were identified from the gene activity markers (Fig. 8B and C). The normalized accessibility, peaks, and co-accessible links of the gene *SNAP25* are shown in Fig. 8D. The script for this analysis can be found in the companion GitHub repository (see the section "Availability of data and software code" for details)

### Single cell gene network analysis

Gene regulatory network (GRN) inference hypothesizes that the etiology of a complex genetic disease is driven by complex signaling cascades [301] and aims to disentangle such signaling maps from molecular data and identify dysregulated subnetworks putative regulators underlying the diseased tissues [302]. GRN inference has been successfully applied to understand complex diseases, including asthma, cancer, flu infection, and neurodegenerative diseases [303–308]. However, GRNs from widely populated bulk sequencing data are limited in resolving inter-wined signaling across and within mixed cell populations, although diseased tissues are composed of heterogeneous cell populations with different morphology and functions [8, 302, 309, 310].

To this end, scRNA-seq has recently emerged to uncover cell-level signaling pathways associated with neurodegenerative diseases and their markers [7, 8, 26, 42, 106, 168, 187, 194], and identify key signaling pathways encompassing distinct cell types [7, 8, 130]. However, data-driven network models of the AD cell types and their regulatory mechanisms have been relatively under-explored. To mitigate this gap, we review the current GRN inference methods in scRNA-seq to evaluate their applicability in different contexts, and recommend workflows to construct robust and accurate network models in AD. While we acknowledge several excellent reviews in the literature [302, 311–313], we notice that the repertoire of reported methods and categories, including existing bulk-based methods, are still evolving to address arising challenges in the field. Thus, we have structured this review section to list the challenges in scRNA-seq GRN inference, the applicability of established bulk-based methods to infer single-cell GRNs, and currently available scRNA-seq-based GRN inference methods adopting different statistical frameworks. We will further address GRN evaluations and recommend workflows to ensure the discovery of robust network models.

#### Challenges in scRNA-seq GRN inference

GRN inference in scRNA-seq is presented with challenges to construct robust and reproducible network models. Drop-out reads present false zero-expressions in addition to the sparse cell-wise transcriptome. These noises in scRNA-seq underscored near-random performances of gene-gene similarity measures to uncover meaningful gene interactions in benchmark scRNA-seq data [314]. Model-based dropout imputation methods such as SAVER [81] have shown outstanding imputation performances with the least false-positives [315], and improved the GRN performances [316]. The high-dimensionality of scRNA-seq with thousands to hundreds of thousands of cells can incur ‘curse-of-dimensionality’ and increase the computational complexity to evaluate gene-gene interactions. Careful feature selection by gene dispersion [74] and low dropout rate [317], and cell selection by low mitochondrial rate, read depth, and number of expressed genes [74, 92] can improve network performances. Further, adjustments for technical variations such as batch effects by the mutual nearest neighbor (MNN) [91] and canonical correlation analysis (CCA) [92] are beneficial for GRN inference [316].

#### scRNA-seq application of bulk-based gene network construction methods

Established bulk-based GRN inference tools have been applied in scRNA-seq as these tools constructed robust network models in complex diseases and are immediately accessible. While there are several excellent reviews on bulk-based GRN inference methods [318–320], we review several established methods applied in the scRNA-seq domain.

Co-expression networks identify gene interactions by gene pairwise association measures such as correlations or information-theoretic measures [321]. Weighted gene co-expression network analysis (WGCNA) is the most popular correlation-based method to construct a scale-free gene interaction network model, and identify co-expressed gene modules as putative interactomes [322]. Multiscale Embedded Gene Co-expression Network Analysis (MEGENA) embeds most correlated gene pairs on a topological sphere to construct a sparse co-expression network and detect multi-scale gene modules [323]. While these correlation-based methods capture linear patterns, information-theoretic measures can capture non-linear patterns. Algorithm for the Reconstruction of Accurate Cellular Networks (ARACNE) is an information entropy-based network inference method and prune false-positive interactions by testing all trios with data processing inequality [324]. CLR is based on mutual information to handle gene-gene interactions and controls false-positives by using the global network as the background [325]. MRNet combines both criteria in CLR and ARACNE to screen the false-positives to improve the prediction acuracy [326].

Several statistical frameworks infer directed interactions between causal and effector genes in contrast to undirected interactions. Bayesian Network (BN) inference provides a flexible framework for identifying directed interactions in causal cascades and integrating upstream regulations such as genetic variants as prior network [327, 328]. Well-established BN tools include RIMBANet [328] and bnlearn [329]. GENIE3 is a random forest (RF) regression method to infer directed causal relationships and has won the DREAM4 challenge as the best performing network inference method [330, 331].

Applications in scRNA-seq have discovered key pathways and markers of heterogeneous cell populations underlying human disease tissues. WGCNA has been applied to identify pathways to activate dormant neural stem cells [332], regulators of chemotherapy resistance in esophageal squamous cell carcinoma [333], and prognostic markers for prostate cancer [334]. MEGENA has been applied to identify enriched pathways in different astrocytic subpopulations in Huntington disease [335], viral infection-regulated pathways in lung epithelium [336].

On the other hand, naïve applications of bulk-based GRN methods involve several shortcomings. They show lower retrieval of known functional links than those inferred from bulk RNA-seq data [316] and primarily associate the modules to cell types that the intricate pathways within the cells [337]. GENIE3, BN, ARACNE, and CLR in in silico simulated and experimental scRNA-seq data showed poor performance in retrieving true interactions from reference sets (e.g., known protein-protein interactions) with little overlaps across them [338]. Rigorous QC to remove unintended co-variations such as batch effects and lowly expressed genes have improved the bulk-based co-expression networks [316].

#### Single-cell-based network analysis tools

##### Boolean

The Boolean network model simplifies the complex biological pathways into a switch-like process that transits the network change from one state to another [339]. In Boolean networks, a node (i.e., gene) is denoted by two possible states, ON (1) or OFF (0), and the interacting relationship between nodes is characterized by a target-node-specific function *f*, which formulates the state of a target gene based on the states of some other genes through clauses consisting of only Boolean operators AND (∧), OR (∨) and NOT (¬) [340]. Several Boolean network methods have been proposed for analyzing scRNA-seq data. Single Cell Network Synthesis (SCNS) [341, 342] is a web-graphic-based tool and uses discretized time series snRNA-seq expression data to infer logical rules driving from early phase to late phase transitions, with single gene change at each transition. The resulting logical model predicts the effects of gene perturbations (e.g., knockout or overexpression) on specific lineages by design. A similar Boolean network method that uses cell trajectory lineage tree information was developed by Chen et al. [343]. Unlike SCNS, BTR (BoolTraineR) [344] does not assume trajectories through cell states. Instead, BTR learns the network structure through iteratively modifying existing Boolean models to explore predictions with an improved match to the observed expression data state via a novel Boolean state space scoring function [344].

##### Differential equation

GRN inference from time-stamped scRNA-seq data can also be facilitated by ordinary differential equation (ODE) models. In these models, a set of ODEs from potential regulators describe temporal changes in the target genes. This model can be expressed in the form of $\frac{dx}{dt}= Ax$, where *x* is a time-labeled vector of *C* single-cell transcriptomic profiles, *x*_1_, *x*_2_, …, *x*_*C*_ ∈ *ℝ*^*G*^ in which *x*_*i*_ represents the expression, for the *i*th cell, of *G* genes, and *A* is a square matrix that characterizes the regulatory network among the genes [345, 346]. One such method, SCODE [346], infers the TF-regulated network by estimating the coefficients of linear ODEs via linear regression in transformed variables. With dimension reduction, this approach leads to a considerable reduction in the time complexity of the algorithm. A similar approach, GRISLI (Gene Regulation Inference for Single-cell with LInear differential equations and velocity inference), was developed [345]. It first estimates each cell’s velocity (i.e., how each gene’s expression value changes as each cell undergoes a dynamical process), then constructs a GRN by solving a sparse regression problem that relates the gene expression and velocity profiles of each cell.

##### Bayesian

A BN inference approach, AR1MA1-VBEM (Variational Bayesian Expectation-Maximization) [347], uses a first-order autoregressive moving-average (AR1MA1) model to fit the fold change of a gene at a specific time with a linear model that combines the data at the previous timepoint and a noise term. Under a Bayesian framework, the likelihood function for the AR1MA1 model is a multivariate Gaussian with mean expressed as a function of the network structure. For ease of computation, conjugate priors are used, and the unknown network structure is modeled as a hidden latent Gaussian variable while a Normal scaled Inverse-Gamma distribution models the parameters of the AR1MA1 model. For actual network inference, it uses a VBEM framework using variational calculus to optimize the network models’ marginal likelihood and posterior distributions. In a different method, HBFM (Hierarchical Bayesian Factor Model) uses a sparse hierarchical Bayesian factor model to formulate the impact of gene expression by various factors associated with each cell, and a gene regulatory network structure is constructed by examining the shared factors between pairs of genes [348].

##### Pseudo-temporal dynamics-based regressions

scRNA-seq data provides an opportunity to estimate the cell-level temporal dynamics by assuming gradual changes in the cellwise transcriptome occurs over time and constitute a trajectory. While cell trajectory inference is an active research area in the scRNA-seq domain [106, 163, 194], the inferred ‘pseudo-time’ on individual cells opens the doors to identify causal expressions in cells from preceding time points to explain downstream changes in the later time points, thus enables inference of causal networks. Granger’s causality is a regression-based framework to explain variations at a lagged time point with several precedent time point data [349] and has been adopted in several scRNA-seq GRN inference methods. SINCERITIES (SINgle CEll Regularized Inference using TIme-stamped Expression profiles) assumes such time-stamped cell transcriptome. Sufficient temporal changes between two ‘snapshots’ of single-cell transcriptome are evaluated by Kolmogorov–Smirnov (KS) statistic, and Granger’s causality infers the causal TF activities to the target genes’ expression changes [350]. To address irregularities in inferred pseudo-time that the underlying dynamical process is not uniform and hence hinders correct causal inference, SCINGE uses kernel-based Granger Causality regression to alleviate irregularities in pseudotime values [351].

Different statistical frameworks have also been adopted to evaluate gene-gene relationships across different time windows. LEAP (Lag-based Expression Association for Pseudotime-series) utilizes Pearson’s correlation of normalized expressions at a time window with those expressions from lagged time windows to establish time-lagged associations between the genes [206]. SCENIC (Single-cell regulatory network inference and clustering) couples co-expressed target genes with TFs by GENIE3 [330] and overlaps them with cis-regulatory binding motifs enrichments within each cell trajectory [352]. SCRIBE uses an information-theoretic measure, restricted directed information (RDI), to quantify the information transferred from the potential regulator to the target in a lagged time point. Qiu et al. showed RNA-velocity, a pseudo-dynamic measure based on transcription kinetics [168], best estimates the real time-series and improves GRN performance over pseudotime [165].

#### Association-based approaches

In contrast to other model-based methods, association-based networks objectively evaluate the likelihood of gene-gene interactions by ‘guilt-by-association’ [320]. Several gene association measures have recently been devised to handle noises and systematic errors specific to scRNA-seq, and optimized algorithms to efficiently dissect robust interactions.

A popular strategy involves a series of data transformation or modeling steps to handle sparsity and dropout expressions and evaluate significant association by correlations or information-theoretic measures. In bigSCale, gene-expressions are grouped into cell clusters [353], and differential expressions between a pair of clusters are transformed into z-scores to calculate Pearson’s correlations between the clusters [354]. With top 0.1% correlations, the global GRN often yields dense networks [311]. CSN (Cell-specific network) [355], on the other hand, aims to infer co-expression networks for individual cells. CSN develops a statistic for each gene pair to evaluate significant patterns in a cell scatter plot, where the statistic is normally distributed if no pattern is present. The significant gene pairs are then collected to construct a cell-specific co-expression network (CSN), and the summarized gene-wise connectivity across cells can serve as denoised and normalized gene expressions for further analyses [355]. Based on multivariate information theory, PIDC utilizes Partial information decomposition (PID) to quantify gene interaction as the proportion of unique information shared explicitly between two genes, compared to the shared information with the rest [338]. When dropout reads were present, PIDC performed favorably over other mutual-information-based methods and yielded sparse GRNs. But, PIDC suffers from data discretization problem, an inherent problem in information-theoretic measures, and is computationally expensive as it sweeps through gene triplets [338]. scLink aims to infer robust and sparse gene-gene covariance structure by modeling the dropout rates per gene to quantify robust expressions. By fitting a Gamma-Normal mixture model for each gene’s expressions, robustly expressed genes are filtered with a low non-detection rate, and the sparse covariance matrix is inferred with a graphical Gaussian model with penalized likelihood method [317].

#### Cell-cell communication network

Cell-cell communication is vital for multicellular organisms to coordinate functionally unique cell populations in response to internal and external stimuli. Such communication is primarily mediated by ligand (L)-receptor (R) interactions and can be visualized by networks where each node is a cell type and the edges are L-R interactions [311]. Several algorithms and databases have been established to leverage cell-level resolution in scRNA-seq to infer cell-cell communication networks, for instance, in cancer research to dissect L-R signaling in tumor micro-environment [356].

iTalk relies on a built-in curated database of 2648 non-redundant and known L-R pairs to infer communications across or within distinct cell types [357]. It models the gain or loss of interactions by differential gene expression of each L-R pair across all cell types independently, given that different cells may have distinct receptors for the same ligand and vice versa [357]. Instead of relying on differential expressions, Zhou et al. focus on highly expressed L and R genes in sender and receiver cells and evaluate their communications by pairwise Spearman correlation [356]. As a single L-R pair can function in multiple cell type pairs, scTensor models cell-cell communication as a hypergraph where each node is a cell type, but the edges represent different related L-R pair sets. This “many-to-many” model of communications across multiple cell types is detangled by non-negative Tucker decomposition to estimate contributions from the expression patterns of receptors and ligands as well as L-R interacting pairs [358]. Different from other methods discussed, SoptSC considers the signaling-pathway-wise gene expressions downstream of each L-R interaction to infer the L-R activity. The signaling probability is defined based on weighted co-expression of pathway activity in the sender-receiver cell pairs. Together with pseudotemporal information inferred from scRNAseq, SoptSC allows inference of higher-level communication networks with more complex structures such as feedback/feedforward interactions [359].

#### Context-based network inference

ACTION attributes functional similarity to genes with relatively weak but preferential expression in specific cell types. It identifies cell clusters, termed archetypes, by low-dimensional geometric constructs in the functional space. Further, it infers cell-type-specific TF regulatory networks (TRNs) by assessing significant TFs with their targeted top-ranked cell type markers. Thus, ACTION provides functional annotation and subsequently the phenotype associated with each cell type [360]. SCINET extends the concept of ACTION to project single-cell transcriptomic data onto a reference interactome and identifies cell-type-specific and disease-associated interactions. By using regression-based imputation and rank-based inverse normal transformation, SCINET infers the likelihood of co-expressed gene-gene interactions, assuming the standard normal distribution of the transformed expression data [361].

#### Network evaluation

The confidence of the inferred interactions is often crucial in identifying key mechanisms and putative regulators, and ultimately, formulating robust and testable biological hypotheses. One approach is to examine the congruence between the established regulatory pathways or relationships and the inferred networks. Gene perturbation databases in established disease models such as Library of Integrated Network-based Cellular Signatures (LINCS) [362] or CREED [363] curate differentially expressed gene signatures by genetic or chemical perturbations in cell lines, animal models, or disease tissues. These signatures represent the downstream pathways of the exerted perturbation on a network regulator, and the topology of the context-matched, inferred networks should capture these pathways within the perturbed regulator’s proximity. Key Driver Analysis (KDA) examines the enrichments of the perturbation signatures in the network neighborhood of the perturbed regulator by Fisher’s Exact Test [307]. The reproducibility of curated reference networks such as protein-protein interactions (PPI) [364], signaling pathways, and text-mining from published studies provide other measures to evaluate inferred networks’ biological relevance. Although incomplete and high in false positives [318], enrichment for the references is a useful metric for evaluating network performance [314]. Perturb-seq provides a single-cell sequencing platform leveraging Cas9/CRISPR to capture the effects of gene perturbations in a high-throughput manner at a cell-level resolution [365].

On the other hand, simulation studies provide the full controls over the model and noise parameters to generate tailored data, whose underlying reference network is known for objective comparison with inferred networks [331]. GeneNetWeaver is a stochastic differential equation-based simulation tool and has been used to evaluate bulk-based GRNs such as DREAM4 challenge [366]. Chen and Mar generated simulated data to resemble scRNA-seq with a combined approach of GeneNetWeaver and added artificial dropout events to evaluate bulk-based, and single-cell-based GRN approaches [367]. Pratapa et al. introduced BEELINE as a single-cell transcriptome simulation framework leveraging Boolean network models [207]. Unlike GeneNetWeaver, BEELINE can simulate stochastic data with underlying cell trajectory, a hallmark feature in single-cell transcriptomes [207].

#### Recommended workflow & applications to AD

Inference of robust and accurate network models in single-cell transcriptome requires appropriate modeling of data noises specific to scRNA-seq. These involve low per-cell coverage, doublets, dropout reads as the main sources of noises and should be handled as illustrated in QC guidelines and impute the dropout reads with model-based methods. The model-based imputations infer cellwise dropout rates to facilitate the selection of cells robustly expressing each gene to improve the performance of inferred gene-gene interactions [317]. In addition, unwanted sources of variations such as batch effects should be adjusted before gene-gene similarity calculation for improved prediction accuracy [316].

Depending on the type of an inferred GRN, the expression data should be adequately normalized to optimize the accuracy of inferred gene interactions. The denoised counts should be log-transformed to closely follow Gaussian distributions for constructing regression-based or association-based GRNs. On the contrary, kinetic-based methods such as Boolean or ODE-based networks may perform better in the gene count space.

However, GRN inference with scRNA-seq data is still in its infancy and requires an objective performance evaluation. BEELINE [207] and GeneNetWeaver with artificial dropout events provide accessible platforms to generate simulated data mimicking scRNA-seq characteristics and to build up respective reference networks. The simulated data should closely follow noise models from the real-world scRNA-seq data, including branching trajectories and match the dropout rates, and an inferred GRN should be compared to the respective reference network for reproducing network connectivity. In addition, the inferred GRNs can be evaluated in biological contexts as enrichment for protein-protein interactions (PPI) or known pathways.

**Fig. 9:**
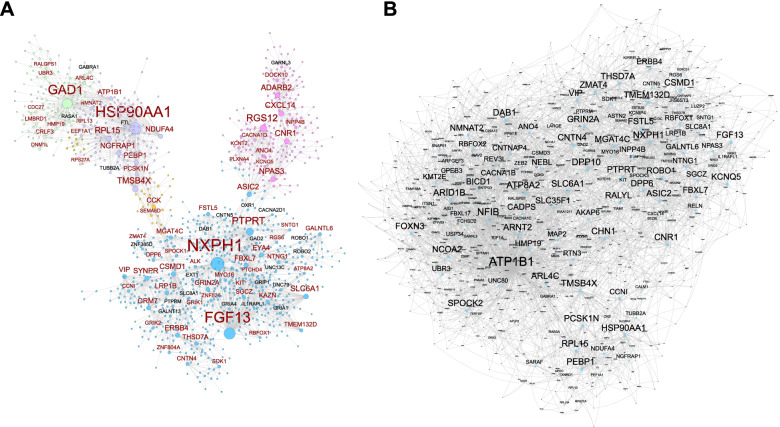
Excitatory neuron cluster-specific gene networks inferred from the ROSMAP AD snRNA-seq data. **A** Coexpression network constructed by MEGENA, with node color denoting the module assignment. **B** Bayesian network constructed by RIMBANet. Node size is proportional to the degree of connectivity

To illustrate the application of network analysis in AD single-cell data, we constructed gene co-expression and Bayesian networks for an excitatory neuron cluster identified in the ROSMAP snRNA-seq data using two well-established bulk-tissue gene network inference tools. Figure 9 visualizes the two networks. The scripts for the network analyses are provided in the companion GitHub repository (see the section "Availability of data and software code" for details) .

### Prioritization of cell clusters

scRNA-seq enables accurate classification of individual cells by gene expression profiles, and cluster-based differential gene expression analysis of scRNA-seq data can help resolve brain-region specific changes in heterogeneous cell populations in AD brains. As loss of neurons and increased microglia activation are characteristic of typical AD brains [368, 369], and many genes show differential expression between AD and control [307, 308, 369, 370], cell clusters from scRNA-seq can be prioritized for their relevance to AD or other diseases by considering the following criteria: 1) the change in the proportion of the cells in a cluster between control and disease (e.g., AD) and 2) the number of DEGs in a cluster between control and disease.

For example, Olah et al. used scRNA-seq to characterize differences in the distribution of distinct microglia subpopulations in human cerebral cortex specimens [7]. The study identified nine distinct sequence profile clusters indicative of 9 distinct microglia subpopulations. Among these, they found reduced frequency of the cluster 7 microglia sub-population in AD brain tissues [7]. Moreover, they found cluster 7 is particularly enriched for genes depleted in the AD cortex [7]. As such, these observations would justify prioritizing microglia cluster 7 as a target for follow-up investigations.

In another single-nucleus transcriptomic study of human prefrontal cortex specimens, Mathys et al. identified transcriptionally distinct sub-populations across six major brain cell types, with many of the top DEGs recurring across multiple cell types [8]. Interestingly, the expression changes of myelination-related genes across major cell types, including oligodendrocytes and oligodendrocyte progenitor cells, are indicative of major perturbations in myelin integrity in AD brains. Thus, combined analysis of specific cell subpopulations and DEGs could help illuminate functional and dynamic changes at the single-cell level between AD and control and provide the basis for prioritizing specific clusters for follow-up functional, mechanistic investigations and therapeutic development.

### Integration of snRNA-seq and bulk RNA-seq datasets

In the past decade, bulk RNA-seq datasets have been massively accumulated. However, cell type compositions in the bulk tissues represent a significant confounding factor influencing sample comparisons. scRNA-seq can be utilized to estimate cell fractions of the bulk RNA-seq datasets, thus ‘*deconvolving*’ the cell compositions. Early deconvolution approaches rely on cell markers and gene expressions from cell sorting experiments. For example, CIBERSORT applies support vector regression to characterize the cell composition of complex tissues from their gene expression profiles [371], and CellMix is an R package that incorporates multiple deconvolution methods (e.g., the Digital Sorting Algorithm and semisupervised non-negative matrix factorization methods (ssKL and ssFrobenius)) to analyze heterogeneous samples [372]. More recently, deconvolution methods have been developed to utilize single-cell transcriptome as the reference directly.

In contrast to traditional methods that mainly use marker genes from cell sorting experiments, single-cell-based deconvolution methods utilize sparse gene expression matrix from scRNA-seq and models gene/sample specific variations unique to single-cell experiments. For example, DeconvSeq utilizes a generalized linear model in feature quantification to construct a projection matrix and resolves cell type fractions by a sequential quadratic programming based solver [373]; MuSiC applies a weighting scheme to prioritize consistent genes across subjects and proposes a weighted non-negative least squares (W-NNLS) regression framework to estimate cell fractions [374]; DWLS designs a weighted least squares approach to adjust the contribution of each gene and solves the constrained dampened weighted least squares problem by quadratic programming [375]; Bisque performs a bulk gene expression transformation to explicitly account for the gene-specific variations and employ the non-negative least-squares (NNLS) regression for cell type fraction inference [376]; and SCDC leverages multiple scRNA-seq reference sets for bulk gene expression deconvolution [377]. Extending from CIBERSORT, CIBERSORTx can also use single-cell expression references to infer cell type abundance and cell-type-specific gene expressions. In AD studies, both traditional and scRNA-seq based methods have been applied to deconvolve the cell fractions of bulk RNA-seq datasets [376, 378]. However, the correlations of the inferred cell fractions with immunohistochemistry (IHC) as ground truth were not high [378]. A more recent study showed that Bisque could reliably estimate cell fractions in subcutaneous adipose and dorsolateral prefrontal cortex expression datasets [376]. When applied to the ROSMAP AD snRNA-seq dataset, Bisque outperformed other deconvolution methods [376].

#### Recommended workflow and applications to AD

**Fig. 10:**
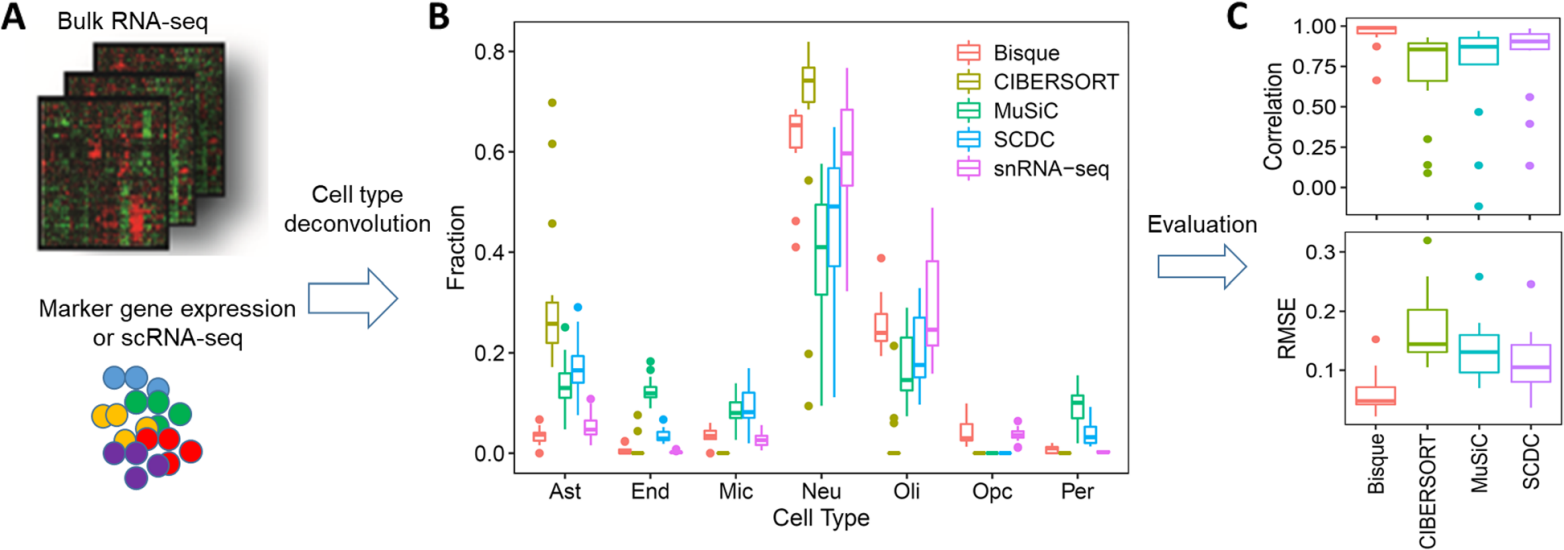
Cell-type deconvolution of the bulk RNA-seq data in the prefrontal cortex in the ROSMAP AD cohort. **A** The input of cell-type deconvolution analysis. The cell-type marker gene or scRNA-seq expression matrix was used to infer cell type fractions of the bulk RNA-seq datasets. **B** Cell-type fractions from the deconvolution analysis of 15 individuals from the ROSMAP database. Four different deconvolution methods were applied, including bulk RNA-seq based method (CIBERSORT) and single-cell-based methods (Bisque, MuSiC, and SCDC). The pink color indicates the cell type fractions estimated by the snRNA-seq experiment. **C** Evaluation of different deconvolution methods by comparing with the cell-type fractions from the snRNA-seq experiment. Two matrices were used to benchmark the tool performance: the Pearson correlation (top panel) and the root-mean-square deviation (RMSE, bottom panel)

Leveraging the ROSMAP AD cohort with both bulk RNA-seq data for over 600 subjects and snRNA-seq data for a subset of the samples, we performed a mini-benchmarking of the performance of several deconvolution methods. Here we aggregated the cells of each individual in the snRNA-seq data by major cell types to calculate a cell type proportion estimate. As shown in Fig. 10, the single-cell-based methods including Bisque, MuSiC, and SCDC tend to have a higher correlation with the snRNA-seq cell type proportions and a lower error rate than the traditional marker gene-based method CIBERSORT (Fig. 10). Among different single-cell-based methods, Bisque’s estimates of the cell type proportions best resemble the results derived from the snRNA-seq experiments. Therefore, single-cell-based methods, especially Bisque, are recommended for the general purpose of deconvolution studies. The script for this integrative analysis is provided in the companion GitHub repository (see the section "Availability of data and software code" for details).

The cell-type proportions estimated from deconvolution methods can be used for a number of downstream analyses for AD. For example, in contrast to traditional DE analysis confounded by cell type proportions, deconvolution results can provide new insights into the cell-intrinsic DEGs by adjusting cell type proportions [379]. The fractions of neuron cells calculated by different deconvolution methods negatively correlated with the cognitive diagnosis [376], consistent with our knowledge of neuronal loss in AD brains. Aneal et al. developed a new deconvolution method CelMod to infer proportions of 35 cell subclusters [380], which were identified by snRNA-seq of 24 prefrontal cortex samples with or without AD pathologies. The deconvolution method revealed that highly correlated cell subclusters form distinct cellular communities across 640 individuals. Although biological functions of the cellular communities need validation, the deconvolution analysis provides intriguing applications in AD studies to analyze interactions among different cell subtypes.

### Prioritization of gene subnetworks and key drivers

Established strategies for prioritizing co-expressed modules and key causal drivers from bulk transcriptomic data can be directly applied to cell cluster-based coexpression and causal networks [307, 328, 370, 381–383]. Specifically, for each cell cluster from scRNA-seq, the modules in the respective coexpression network will be tested for and sorted by associations with trait phenotypes. The association between module eigengenes (the first principal component of the module gene expression data) and the biological covariates can be utilized to evaluate association [384]. Alternatively, the modules can be tested for enrichment of DEGs in the cluster (see Differential expression for disease gene identification section) between disease (e.g., AD) and control, and then all the modules will be rank-ordered by enrichment score (e.g., FET *p*-value or fold enrichment) [385]. Similarly, the network neighborhood of a gene conforms to the model of the genes’ downstream pathways, and the enrichment of cluster-specific DEGs from diseased cells (e.g., AD) in the network neighborhood can guide the prediction of key drivers of the disease etiology [386]. Such key driver prediction methods have been widely used in numerous network analyses of human diseases, such as AD, coronary artery disease, inflammatory bowel disease, and allograft rejection [307, 386–390]. Key drivers are thought to provide insights into gene regulatory control and serve as targets for therapeutic discovery. Key drivers then can be prioritized for experimental validation by considering a line of evidence whichever available and appropriate, including but not limited to the degree of connectivity, the status of expression change between disease and control, fold enrichment of disease associated genes in the downstream subnetwork, strength of expression correlation with disease trait variables, genetic association signal, literature support, etc. [387].

### Spatial transcriptomics

Cellular spatial location is not well retained in the single-cell transcriptomics methods described above. Given that cellular gene expression patterns change in response to environmental cues maintaining the neighborhood environment allows for a deeper understanding of tissue-wide dynamics and biological function of genes and cells. Many of the current spatial technologies were derived from RNA in situ hybridization (ISH) and/or principles of laser capture microdissection. While ISH, a commonly used technique to histologically visualize mRNA localization in tissues on microscope slides, allows for maintained tissue integrity and entire tissue analyses, a major limitation of traditional ISH is the number of targets that can be analyzed concurrently. On the other hand, laser capture microdissection, developed in the 1990s, uses low-power infra-red laser beams to microdissect cells of interest to overcome this obstacle and allows the whole transcriptome profiling, but the laser capture microdissection is destructive for the cells.

Several advanced approaches have been developed to profile whole transcriptomes while preserving spatial information in the past few years. These include fluorescence in situ hybridization (FISH) [391–396], in situ sequencing (ISS) [397–399], and spatially-barcoded RNA sequencing [23, 400–402]. The methods differ in sequencing depth, the number of transcripts analyzed (dozens to the whole transcriptome), tissue integrity (dissociative versus non-destructive), cellular throughput (10s, 100 s, 1000s of cells), spatial information (image or spatial barcode), cellular resolution (multi, single or subcellular) and starting material (fixed or frozen tissue).

These technologies open an unprecedented opportunity to dissect the cellular complexity and characterize the tissue microenvironment for identifying inter-cellular interactions and signaling pathways in complex diseases such as cancer [201, 403], amyotrophic lateral sclerosis (ALS) [404], and AD [405]. Computational methods have been developed to identify spatial patterns [406–409] and detect spatial ligand-receptor interactions [406]. Application of spatial transcriptomics (ST) analysis to mouse models of familial AD has identified two co-expression networks in small tissue domains, representing spatially coordinated transcriptomic changes induced by amyloid plaques [405].

**Fig. 11:**
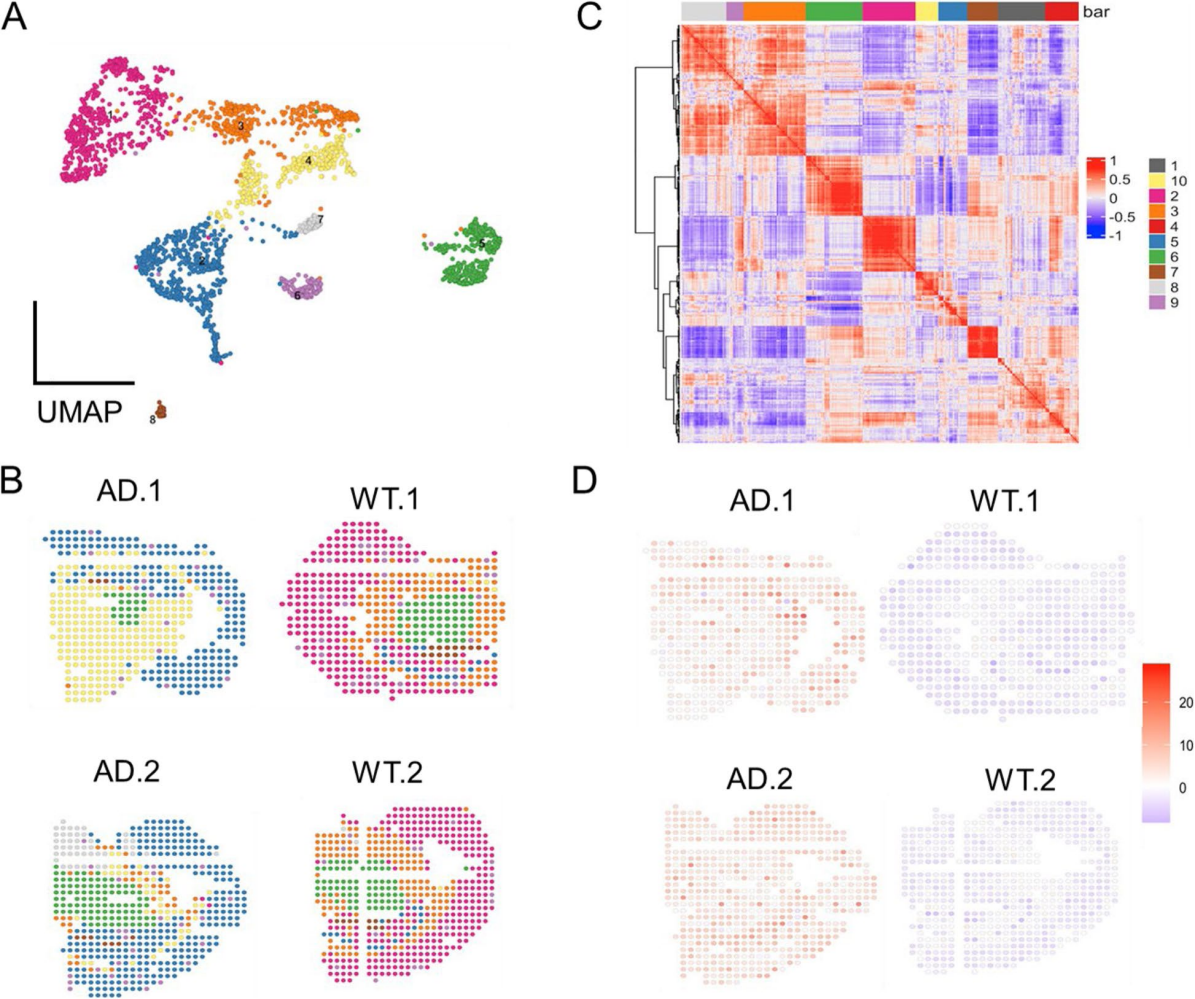
Spatial transcriptomics analysis of an AD mouse model. Data from four 18-month-old mice, including 2 AD (AD.1 and AD.2) and 2 WT (WT.1 and WT.2), were analyzed. **A** Visualization of the Leiden clustering of the tissue domains in the UMAP space; **B** Spatial distribution of the tissue domains colored by the cluster id in **A**; **C** Clustering of spatially correlated genes; **D** Spatial pattern of a spatially correlated gene cluster #6 which is upregulated in the AD mice

Chen et al. [405] applied ST (10X Visium protocol that profiles tissue domains up to ~ 10 cells each) and ISS technologies to study AD using APP^NL-G-F^ mice. They identified two co-expression networks, one of which (referred to as plaque-induced genes, or PIG for short) is particularly interesting since their activities are strongly associated with the Aβ plaque. We applied Giotto [406] to re-analyze a subset of the ST data in their paper, corresponding to two wild-type (WT) and two AD (APP^NL-G-F^) mice at the 18-month-old age. By using Leiden clustering, we identified 8 distinct clusters (Fig. 11A). The spatial patterns of these clusters are robustly reproduced in the normal mice, but vary significantly in the AD mice, suggesting extensive structural differences (Fig. 11B). To quantify the spatial patterns at the gene-level, we used the binSpect algorithm [406] to identify spatially variable genes. The top 300 genes selected by the algorithm were further divided into 20 distinct modules based on co-expression analysis (Fig. 11C). Module 6 is notable as it strongly overlaps with the PIG network previously identified by Chen et al. but a different analysis procedure that requires the knowledge of plaque locations is not used here. Among the 43 genes contained in Module 6, 20 are from the PIG network. These genes are highly expressed in the AD mice but significantly down-regulated in the WT mice (Fig. 11D). We further investigated the cell-type distributions in the normal and AD mice. Since the spatial resolution of the ST technology is limited to 100um, we applied cell-type enrichment analysis to computationally estimate the spatial distribution of different cell types, using publicly available single-cell RNAseq data [410] as guidance. We found that the spatial distributions of neurons are similar, but the proportion of microglia increased significantly in the AD mice. This is consistent with the original study since microglia have been well-known to be associated with AD. Thus, ST analysis provides a valuable tool to identify distinct structural alterations associated with AD and may lead to new hypotheses for future studies. The script for analyzing the spatial transcriptomics data is provided in the companion GitHub repository (see the section "Availability of data and software code" for details).

### Integrative analysis of human and mouse AD scRNA-seq data

As eventually mouse models will be used to validate key findings from human AD single cell sequencing data, integration of mouse and human single cell data is critical for informing the correspondence between AD mouse models and human AD. There are nine papers about single-cell sequencing analysis of AD mouse models [57, 139, 411–417]. As summarized in Table 5, 14 mouse models have been analyzed, including the most widely used 5XFAD, 3XTg-AD, and APP/PS1 transgenic mice. The mouse ages range from 3 to 24 months, and the brain regions profiled include the prefrontal cortex, cerebral cortex, hippocampus, subventricular zone, and cerebellum.

**Table 5:** Summary of mouse models used in single cell sequencing studies of AD

Reference (PMID)	Mouse Model	Brain Region	Age (Month)
32341542	5XFAD	Prefrontal cortex	7, 10
32320664	APP/PS1 IL33 mice, APP/PS1 transgenic mice	Cerebral cortex	10–12
31932797	Trem2_KO mice, Trem2_KO_5XFAD, WT_5XFAD mice	Cortex, Hippocampus	7, 15
31928331	APP23 transgenic mice (B6. Cg-Tg (Thy1-APP)3Somm/J)	Hippocampus	6, 24
29020624	CK, CK-p25	Hippocampus	0wk,1wk, 2wk and 6wk after p25 induction into 3 m-old mouse
28602351	5XFAD	Cortex, Cerebellum	6
32503894	3XTg-AD mice	Subventricular zone and hippocampus	8
32503894	anti-Nk1.1 treated 3XTg-AD	Subventricular zone and hippocampus	7
32579671	5XFAD-CV, 5XFAD-R47H	Cortex and hippocampus	5.5
31902528	Trem2 +/+ mice, Trem2 +/− mice, Trem2 −/− mice	Hippocampus	12–14

So far, few studies have conducted an integrative analysis of human and mouse single-cell transcriptomic data in AD. In a recent single-cell study of TRME2 R62H mutation in AD, Zhou et al. [57] analyzed the human AD snRNA-seq data and mouse scRNA-seq data separately and then compared the cell type-specific disease signatures between the two species by overlapping analysis. However, they fell short of joint learning of single-cell datasets between humans and mice. There are a number of challenges in cross-species single-cell data integration, including but not limited to the partially overlapping gene background, distinct cell populations, and poorly conserved cell type transcriptome or markers that may arise from different species. Nonetheless, some tools have been developed to formally reconcile heterogeneous scRNA-seq data from multiple species to gain robust and insightful comparisons between different species [418, 419]. Assuming that at least a subset of gene-gene correlations should be conserved, thus align cells across species, Butler et al. [49] proposed to first use canonical correlation analysis (CCA) to identify conserved gene-gene correlation structure between datasets and then apply nonlinear warping algorithms to align different datasets for a single integrated clustering analysis. When applying this alignment procedure to single cell datasets from human and mouse pancreatic islets, the integrative analysis was able to identify conserved cell states and cell-type markers, with cluster calls agreed very well (> 94%) with the analyses from the independent data sets [49]. In theory, other batch correction pipelines based on similar assumptions, such as MNN [91] and Harmony [98], can also be used for cross-species joint learning. Different from the joint clustering in CCA, Crow et al. developed a supervised framework to align cell clusters across datasets in their method MetaNeighbor [420]. In this method, each dataset is first analyzed separately to label the cell types. Then a cross-dataset cell correlation network is constructed based on the expression of a given set of genes. Next, the cell-type labels (“identity”) in one dataset are held-out and predicted using a neighbor-voting classification model trained from the remaining cell correlation network in other dataset(s). This training and prediction process iterates for every dataset. This method can rapidly evaluate how well cell types are conserved/replicated in different conditions by comparing the predicted labels with original labels from individual analyses. MetaNeighbor has been successfully applied to compare cell atlases across 7 species [418].

**Fig. 12:**
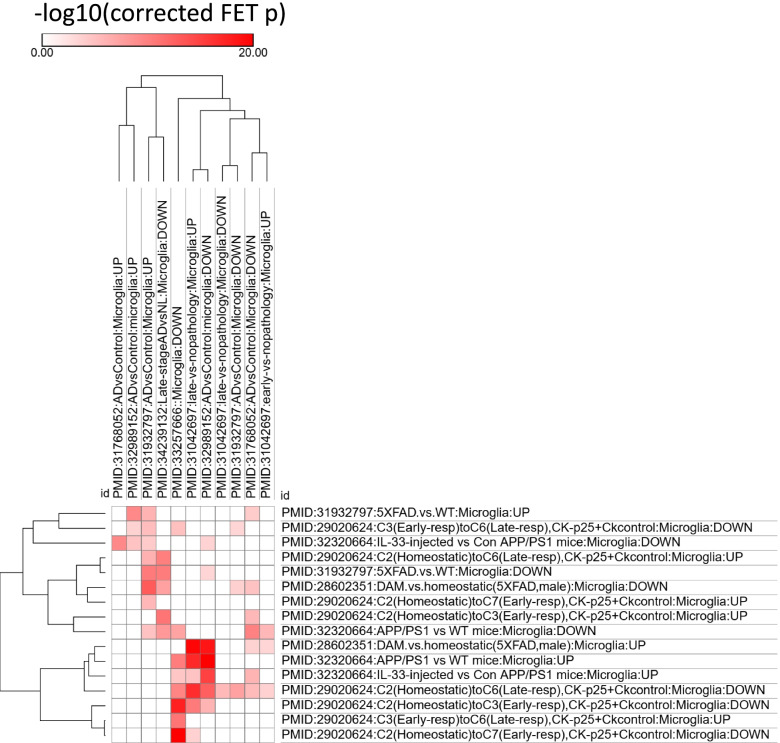
Heatmap showing the overlap of microglia specific DEG signatures between human and mouse AD single cell datasets. Each row represents a mouse study. Each column represents a human study

To perform a comprehensive comparison of single cell-based gene signatures between human AD studies and AD mouse models, we collected cell-type-specific disease signatures from six single-cell AD mouse model studies [57, 139, 414–417] and seven single-cell human AD studies [7, 8, 41, 42, 45, 57, 421]. Mouse genes were converted to human homologs by using BioMart before enrichment analysis by FET. We compared the DEG signature for each cell type. In microglia, the DEGs from 5XFAD, APP/PS1, and the CK-p25 mice show the most significant enrichment with human microglia DEGs (Fig. 12). Yet, no single mouse model can capture all molecular alterations in human AD, and the selection of mouse models for signature validation may be cell type-dependent.

### Challenges in using human postmortem and mouse tissues

There are multiple challenges in using human postmortem and mouse tissues to study AD due to the heterogeneity of human samples and human–mouse discrepancies. First, the existing bulk- or single cell/nucleus-based RNA-seq data from human postmortem brain tissues in AD are from terminal-stage patients with extensive neuronal cell death, a prolonged process starting already at preclinical stages before any clinical symptom emerges [368, 422, 423]. However, terminal-stage tissues may not inform the progression of pathogenic mechanisms of earlier disease stages [424, 425]. Mouse tissues, on the other hand, often encapsulate earlier stages of the disease characterized by accelerated amyloid or tau proteinopathy [424]. Second, molecular changes like stress-induced inflammatory response may occur postmortem should cellular energy stores and temperature permit [425], leading to augmented transcriptomic changes that confound the real biological signal. A recent transcriptomic study of brain tissues found rapid loss of neuronal genes and reciprocal expression of glial genes during a postmortem interval (PMI) period of 24 h [426]. Such postmortem changes may lead to loss of cell identity, which in turn biases the subsequent cell clustering. Cautions need be taken when examining cell clusters, especially, neuronal clusters with weak markers expression. Genome-wide modeling of RNA fidelity as a function of PMI is necessary to alleviate such impact and help determine and correct the gene expression pattern for more robust cell clustering and downstream analyses. Therefore, compared to mouse brain samples, human brain samples likely present more variable and poorer RNA quality due to PMI effects [424, 427]. Finally, differences in tissue collection and processing may also confound sequencing data. For example, there are differences in gene and cell type coverages between scRNA-seq and snRNA-seq data as reviewed above. Given current technological and ethical constraints, snRNA-seq analysis is more practical than scRNA-seq for human postmortem brain tissues. However, we must note that snRNA-seq is limited for detecting cellular activation in microglia in human diseases [31], a potential explanation for inconsistent observation of microglia signatures between human studies and between human and mouse studies.

While high quality mouse biospecimens are more readily available, it remains a challenge to choose appropriate AD mouse models (familial AD versus sporadic AD [428]), with a proper experimental design taking into consideration of critical factors including age, sex, brain regions, sample sizes, etc. AD-like pathology occurs in different brain regions of varying mouse models at different developmental stages [429–431]. For example, rTg4510 mice show tauopathies in the cortex at 4 months, while the hippocampus at 5.5 months [430]. The PS19 mice show neuron loss in the hippocampus at 8 months old, which spreads to other regions by 12 months. Existing mouse-based single-cell studies of AD have used the prefrontal cortex, cerebral cortex, hippocampus, subventricular zone, and cerebellum for scRNA-seq analysis (Table 5). Some of the brain regions used in human single-cell sequencing studies have not yet been profiled in mice (e.g., the entorhinal cortex and superior frontal gyrus). Lastly, as the existing AD mice mimic some, but not all the key pathologies of human AD, better preclinical models of AD are urgently required.

### Future development for bioinformatics of single-cell sequencing data

#### Outlook of cell clustering analysis in AD scRNA-seq

Cell clustering in scRNA-seq is mostly performed within widely adopted scRNA-seq workflows such as Scater [74], Seurat [92], SCANPY [50] and monocle [432]. However, cell clustering is typically dependent on dimension reduction in these workflows and offers a limited range of clustering algorithms (e.g., kNN-based Louvain’s algorithm). On the other hand, much of the recent developments in cell clustering remained untapped in scRNA-seq study of AD. For instance, the application of genotype-based clustering approaches to dissect cell populations expressing pathogenic variants exemplifies such untapped potentials. Deep learning-based approaches are also attractive, scalable alternatives to extract non-linear patterns in massive AD single-cell transcriptome while seamlessly handling batch effects and scRNA-seq specific noises. In addition, we anticipate single-cell-based spatial transcriptomics to become available in the near future. Then novel clustering methods are needed to leverage the spatial information loss in the current single cell data to bring in the potentially important topological context in cell clustering analysis. Overall, the advances in cell clustering will expand the repertoire of AD-associated cell populations, thereby enhancing our understanding of underlying cell type-specific mechanisms.

#### Suggestions for future trajectory inference approaches

While several dimension reduction methods such as PCA, t-SNE [101], DiffusionMap [102] and UMAP [103] have been widely adopted in the scRNA-seq analysis [34], some methods are not optimized for dissecting cell trajectories. Non-linear projection methods such as t-SNE and UMAP are known to distort the underlying data structure while mapping cells to low-dimensional manifolds discards long-range structures, and their stochasticity yields slightly different results if the seed is not properly initialized. On the other hand, Diffusion Map reflects local and long-range structures and is optimized to trace gradual changes in the transcriptome, making it an attractive tool for trajectory inference [102]. However, diffusion maps are computationally expensive, and PCA can serve as an efficient alternative to capturing long-range structures to identify the spanning trajectories in large-scale scRNA-seq data.

The surge in trajectory inference method development urgently needs appropriate scenario-specific methods. A growing effort has been put into this direction [433, 434]. A recent comparative study benchmarks 45 trajectory inference methods over hundreds of simulated and real-world data and offers a set of guidelines to walk users through method selection [313]. Notably, users should be aware of whether the trajectory structures can be pre-defined and fit the current experimental settings. Determination of the starting point often requires manual selection based on prior knowledge or marker gene expression. An unsupervised method using the quantile polarization of a cell’s principal component values has been recently proposed and subsequently validated in several independent studies [435]. The scalability and usability should be considered for the efficient characterization of large-scale single-cell data. Most importantly, trajectory inference results should be seen as hypotheses that need validation regarding their applicable scenarios, robustness, and noise tolerance.

#### Future directions of sc-CNV and sc-eQTL analysis for AD

Even though single-cell based CNV and sc-eQTL analyses have been primarily performed in cancer researches [9, 211, 212] and human-induced pluripotent stem cell studies [261, 268], single-cell based genetic variation analysis hasn’t been applied to AD. So far, there are about 50 eQTL studies [244, 436–482] and about 30 CNV studies [483–515] in AD, based on bulk tissues only. Cell-type specific and brain region-specific genetic mutations have been shown more relevant to the pathology process of AD [244, 308, 450, 452, 457, 470, 516]. For example, some sc-eQTLs are cell-status dependent [247, 260]. Additional future study subjects include the functional differences of eQTLs between the normal aging process and pathologic process in AD, and the relevance of cell type-specific eQTL with respect to ApoE or Tau status. Single-cell-based genetic variation analysis can pinpoint key genetic mutations driving the pathological progress of specific cell populations such as microglia cells and neurons in AD. As AD is heterogeneous at both pathological and transcriptomic levels [6], it would be interesting to understand how genetic variations drive AD heterogeneity. Single-cell genetic variation analysis may offer a novel avenue to understand the genetic heterogeneity of AD using single-cell genetic variation analysis.

#### Drug development for AD using single-cell sequencing data

Single-cell sequencing can identify key molecular pathways targets from distinct cell types in AD and resolve the mixed signals from bulk tissues. Particularly, cell-type-specific signatures from scRNA-seq can be useful for repositioning FDA-approved drugs for treating AD. Through reversal of the cell-type-specific signatures, Connectivity map (CMap) and LINCS have been used to predict candidate FDA-approved drugs for several diseases, including pulmonary arterial hypertension (PAH) [517], COVID-19 [518] and lymphangioleiomyomatosis [519]. For instance, Hong et al. identified NF-κB signaling upregulation and IFN signaling downregulation in several cell types of PAH using scRNA-seq and applied the signatures to the CMap predicting candidate drugs to reverse the changes. Our group developed a more accurate algorithm [520] to identify the CMap and LINCS compounds that reverse the cell-type-specific signatures precisely.

#### Experimental validation of novel cell subpopulations associated with AD

As high-throughput sequencing techniques produce numerous data and hypotheses, additional experimental validation is often required to confirm the findings. Several scRNA-seq studies have revealed and experimentally validated AD-associated gene regulation and cell subpopulations with a primary emphasis on the glial cells [7, 57]. Co-immunostaining of a cluster-specific signature gene with a known general cell type marker provides the most straightforward visualization and quantification of each cell subpopulation. However, it often depends on the antibody specificity and availability of the marker genes of interest and is generally low throughput. Alternatively, RNAscope, an in-situ hybridization (ISH)-based multiplexing method with high target-detection specificity, can be applied if no working antibody is available or multiple signature genes are required to define a subset of cells [405]. Other validation strategies include the NanoString nCounter system to verify cluster-specific signature gene expression and cross-validation in independent cohorts with cell cluster alignment [49]. In summary, experimental validations from various angles would greatly enhance our confidence in identifying novel cell subpopulations associated with AD and serve as the basis for targeted therapeutic development.

## Conclusions

In conclusion, we comprehensively reviewed the state-of-the-art bioinformatics approaches to analyze single-cell sequencing data and their applications to AD in 14 major directions. The basic analyses include data quality control and normalization, cell cluster identification and cell subpopulation characterization and differential expression while more advanced analyses involve trajectory inference, copy number variation, eQTL identification, and integrative gene network inference. We also reviewed the recent progress on analyzing scATAC-seq and spatial transcriptomics data and integrating single-cell multi-Omics data. We summarized their advantages and disadvantages for multiple methods in each direction to help users select the most appropriate approach for specific applications. More importantly, we have implemented the recommended workflow for each major analytic direction and applied it to an snRNA-seq dataset in AD while the scripts and the data are shared with the research community. We further discussed the potential future development of bioinformatics of single-cell sequencing data. We expect both less experienced and advanced single cell data analysts would be greatly benefited from the review and the accompanied software tools. As such, this review not only provides insights into various methods to analyze scRNA-seq data and guidelines for analyzing AD scRNA-seq data but also serves as an invaluable resource for the AD research community and the single cell sequencing community in general.

## Supplementary Information

**Additional file 1: Supplementary Table S1.** The chr19 amplification region inferred in the Cluster 6 of late-pathology AD cases. **Supplementary Table S2.** Genes in the chr19 amplification region in the Cluster 6 of late pathology ADs.

## Data Availability

The human postmortem sequencing data are available via the AD Knowledge Portal (https://adknowledgeportal.synapse.org). The AD Knowledge Portal is a platform for accessing data, analyses, and tools generated by the Accelerating Medicines Partnership Alzheimer’s Disease (AMP-AD) Target Discovery Program and other NIA-supported programs to enable open-science practices and accelerate translational learning. The data, analyses, and tools are shared early in the research cycle without a publication embargo on secondary use. Data are available for general research use according to the following requirements for data access and data attribution (https://adknowledgeportal.synapse.org/DataAccess/Instructions). The ROSMAP AD snRNA-seq data analyzed in this study is available at https://www.synapse.org/#!Synapse:syn18681734. The scripts and software tools utilized in this study are available in Github (https://github.com/songw01/AD_scRNAseq_companion).

## Abbreviations

AD: Alzheimer’s disease
Aβ: Amyloid-beta
NFT: Neurofibrillary tangle
CNS: Central nervous system
EOAD: Sporadic early-onset AD
LOAD: Sporadic late-onset AD
MCI: Mild cognitive impairment
scRNA-seq: Single-cell RNA sequencing
snRNA-seq: Single-nucleus RNA sequencing
scATAC-seq: Single-cell assay for transposase-accessible chromatic sequencing
TMM: Trimmed mean of M-values
UQ: Upper-quantile
RLE: Relative log-expression
CNV: Copy number variation
eQTL: Expression associated quantitative trait loci
eCNV: Expression associated CNV
ZINB: Zero-inflated negative binomial distribution
ZINB-WaVE: Zero-Inflated Negative Binomial-based Wanted Variation Extraction
PCA: Principal Component Analysis
t-SNE: t-distributed stochastic neighbor embedding
DM: Diffusion map
UMAP: Uniform manifold approximation and projection
SNV: Single nucleotide variant
kNN: k-nearest neighbor
DCA: Deep count autoencoder
scVI: Single-cell variational inference
WGS: Whole-genome sequencing
WES: Whole-exome sequencing
DE: Differential expression
DEG: Differentially expressed gene
GLM: Generalized linear model
MST: Minimum spanning trees
ICA: Independent component analysis
OU process: Ornestain-Uhlenbeck process
LLE: Locally-linear-embedding
DPT: Diffusion Pseudotime
SNP: Single nucleotide polymorphism
sc-eQTL: Single-cell eQTL
TF-IDF: Term-frequency inverse-document-frequency
LSI: Latent Semantic indexing
SVD: Singular value decomposition
MDS: Multi-dimensional scaling
LDA: Latent Dirichlet allocation
VAF_RNA_: Biallelic polymorphism loci
GRN: Gene regulatory network
CSN: Cell-specific network
PID: Partial information decomposition
TF: Transcription factor
TRN: TF regulatory network
KDA: Key driver analysis
PPI: Protein-protein interaction
PMI: Postmortem interval
