# Supplementary material for: Guidelines for bioinformatics of single-cell sequencing data analysis in Alzheimer’s disease: review, recommendation, implementation and application
Source: Mol Neurodegener. 2022 Mar 2;17:17. doi: 10.1186/s13024-022-00517-z (PMC8889402; doi:10.1186/s13024-022-00517-z)
Supplement: Supplementary file 1 — Additional file 1: Supplementary Table S1. The chr19 amplification region inferred in the Cluster 6 of late-pathology AD cases. Supplementary Table S2. Genes in the chr19 amplification region in the Cluster 6 of late pathology ADs. [file 13024_2022_517_MOESM1_ESM.docx]

**Supplementary Table S1**. The chr19 amplification region inferred in the Cluster 6 of late-pathology AD cases

| **cell_group_name** | **cnv_name** | **state** | **chr** | **start** | **end** |
| --- | --- | --- | --- | --- | --- |
| Ex_late-pathology_s1 | 19-region_21 | 3 | 19 | 571277 | 55403250 |

**Supplementary Table S2**. Genes in the chr19 amplification region in the Cluster 6 of late pathology ADs

| **cell_group_name** | **gene_region_name** | **state** | **gene** | **chr** | **start** | **end** |
| --- | --- | --- | --- | --- | --- | --- |
| Ex_late-pathology_s1 | 19-region_21 | 3 | *BSG* | 19 | 571277 | 583493 |
| Ex_late-pathology_s1 | 19-region_21 | 3 | *PALM* | 19 | 571277 | 583493 |
| Ex_late-pathology_s1 | 19-region_21 | 3 | *GPX4* | 19 | 708935 | 748329 |
| Ex_late-pathology_s1 | 19-region_21 | 3 | *RPS15* | 19 | 1103926 | 1106791 |
| Ex_late-pathology_s1 | 19-region_21 | 3 | *CELF5* | 19 | 1438358 | 1440495 |
| Ex_late-pathology_s1 | 19-region_21 | 3 | *NFIC* | 19 | 3224661 | 3297076 |
| Ex_late-pathology_s1 | 19-region_21 | 3 | *ATCAY* | 19 | 3314403 | 3469217 |
| Ex_late-pathology_s1 | 19-region_21 | 3 | *EEF2* | 19 | 3879864 | 3928082 |
| Ex_late-pathology_s1 | 19-region_21 | 3 | *ZBTB7A* | 19 | 3976056 | 3985463 |
| Ex_late-pathology_s1 | 19-region_21 | 3 | *MAP2K2* | 19 | 4043303 | 4066899 |
| Ex_late-pathology_s1 | 19-region_21 | 3 | *ANKRD24* | 19 | 4090321 | 4124122 |
| Ex_late-pathology_s1 | 19-region_21 | 3 | *SH3GL1* | 19 | 4183354 | 4224814 |
| Ex_late-pathology_s1 | 19-region_21 | 3 | *KDM4B* | 19 | 4360370 | 4400547 |
| Ex_late-pathology_s1 | 19-region_21 | 3 | *PTPRS* | 19 | 4969113 | 5153598 |
| Ex_late-pathology_s1 | 19-region_21 | 3 | *INSR* | 19 | 5158495 | 5340803 |
| Ex_late-pathology_s1 | 19-region_21 | 3 | *PIN1* | 19 | 7112255 | 7294414 |
| Ex_late-pathology_s1 | 19-region_21 | 3 | *ELAVL3* | 19 | 9835257 | 9849689 |
| Ex_late-pathology_s1 | 19-region_21 | 3 | *TNPO2* | 19 | 11451326 | 11481046 |
| Ex_late-pathology_s1 | 19-region_21 | 3 | *MAST1* | 19 | 12699194 | 12724011 |
| Ex_late-pathology_s1 | 19-region_21 | 3 | *NFIX* | 19 | 12833951 | 12874952 |
| Ex_late-pathology_s1 | 19-region_21 | 3 | *CACNA1A* | 19 | 12995608 | 13098796 |
| Ex_late-pathology_s1 | 19-region_21 | 3 | *ADGRL1* | 19 | 13206442 | 13633025 |
| Ex_late-pathology_s1 | 19-region_21 | 3 | *BRD4* | 19 | 14147743 | 14206187 |
| Ex_late-pathology_s1 | 19-region_21 | 3 | *AKAP8L* | 19 | 15235519 | 15332545 |
| Ex_late-pathology_s1 | 19-region_21 | 3 | *MYO9B* | 19 | 15380050 | 15419141 |
| Ex_late-pathology_s1 | 19-region_21 | 3 | *UNC13A* | 19 | 17075781 | 17214537 |
| Ex_late-pathology_s1 | 19-region_21 | 3 | *MAST3* | 19 | 17601328 | 17688365 |
| Ex_late-pathology_s1 | 19-region_21 | 3 | *RAB3A* | 19 | 18097793 | 18151692 |
| Ex_late-pathology_s1 | 19-region_21 | 3 | *JUND* | 19 | 18196784 | 18204042 |
| Ex_late-pathology_s1 | 19-region_21 | 3 | *SSBP4* | 19 | 18279694 | 18281622 |
| Ex_late-pathology_s1 | 19-region_21 | 3 | *FKBP8* | 19 | 18418864 | 18434562 |
| Ex_late-pathology_s1 | 19-region_21 | 3 | *UBA52* | 19 | 18531751 | 18544077 |
| Ex_late-pathology_s1 | 19-region_21 | 3 | *CRLF1* | 19 | 18571730 | 18577550 |
| Ex_late-pathology_s1 | 19-region_21 | 3 | *TMEM59L* | 19 | 18572220 | 18607741 |
| Ex_late-pathology_s1 | 19-region_21 | 3 | *CRTC1* | 19 | 18607430 | 18621039 |
| Ex_late-pathology_s1 | 19-region_21 | 3 | *SUGP2* | 19 | 18683678 | 18782333 |
| Ex_late-pathology_s1 | 19-region_21 | 3 | *HAPLN4* | 19 | 18990888 | 19034023 |
| Ex_late-pathology_s1 | 19-region_21 | 3 | *YJEFN3* | 19 | 19254756 | 19262804 |
| Ex_late-pathology_s1 | 19-region_21 | 3 | *ZNF91* | 19 | 19528861 | 19537581 |
| Ex_late-pathology_s1 | 19-region_21 | 3 | *LSM14A* | 19 | 23304991 | 23395471 |
| Ex_late-pathology_s1 | 19-region_21 | 3 | *APLP1* | 19 | 34172504 | 34229515 |
| Ex_late-pathology_s1 | 19-region_21 | 3 | *CLIP3* | 19 | 35867899 | 35879792 |
| Ex_late-pathology_s1 | 19-region_21 | 3 | *SIPA1L3* | 19 | 36014660 | 36033343 |
| Ex_late-pathology_s1 | 19-region_21 | 3 | *ACTN4* | 19 | 37907208 | 38208369 |
| Ex_late-pathology_s1 | 19-region_21 | 3 | *HNRNPL* | 19 | 38647649 | 38731589 |
| Ex_late-pathology_s1 | 19-region_21 | 3 | *PLD3* | 19 | 38836388 | 38852347 |
| Ex_late-pathology_s1 | 19-region_21 | 3 | *SPTBN4* | 19 | 40348456 | 40380439 |
| Ex_late-pathology_s1 | 19-region_21 | 3 | *ATP1A3* | 19 | 40466241 | 40576464 |
| Ex_late-pathology_s1 | 19-region_21 | 3 | *GRIK5* | 19 | 41966582 | 41997497 |
| Ex_late-pathology_s1 | 19-region_21 | 3 | *CALM3* | 19 | 41998321 | 42069498 |
| Ex_late-pathology_s1 | 19-region_21 | 3 | *ARHGAP35* | 19 | 46601074 | 46610782 |
| Ex_late-pathology_s1 | 19-region_21 | 3 | *SLC8A2* | 19 | 46860997 | 47005077 |
| Ex_late-pathology_s1 | 19-region_21 | 3 | *NAPA* | 19 | 47428017 | 47471893 |
| Ex_late-pathology_s1 | 19-region_21 | 3 | *LMTK3* | 19 | 47487637 | 47515091 |
| Ex_late-pathology_s1 | 19-region_21 | 3 | *CA11* | 19 | 48485271 | 48513935 |
| Ex_late-pathology_s1 | 19-region_21 | 3 | *FTL* | 19 | 48637946 | 48646187 |
| Ex_late-pathology_s1 | 19-region_21 | 3 | *SNRNP70* | 19 | 48965309 | 48966879 |
| Ex_late-pathology_s1 | 19-region_21 | 3 | *SLC17A7* | 19 | 49085419 | 49108605 |
| Ex_late-pathology_s1 | 19-region_21 | 3 | *RPL13A* | 19 | 49429401 | 49442360 |
| Ex_late-pathology_s1 | 19-region_21 | 3 | *LRRC4B* | 19 | 49487554 | 49492308 |
| Ex_late-pathology_s1 | 19-region_21 | 3 | *PPP2R1A* | 19 | 50516892 | 50568435 |
| Ex_late-pathology_s1 | 19-region_21 | 3 | *CACNG8* | 19 | 52190048 | 52229518 |
| Ex_late-pathology_s1 | 19-region_21 | 3 | *MBOAT7* | 19 | 53963040 | 53990215 |
| Ex_late-pathology_s1 | 19-region_21 | 3 | *RPL28* | 19 | 54173412 | 54189882 |
| Ex_late-pathology_s1 | 19-region_21 | 3 | *UBE2M* | 19 | 55385932 | 55403250 |
